# Antioxidant, Anti-Inflammatory and Anti-Diabetic Activities of *Tectona grandis* Methanolic Extracts, Fractions, and Isolated Compounds

**DOI:** 10.3390/antiox12030664

**Published:** 2023-03-08

**Authors:** Mei Han, Fengxian Yang, Kun Zhang, Jiyan Ni, Xia Zhao, Xuelin Chen, Zhennan Zhang, Hanlei Wang, Jing Lu, Yumei Zhang

**Affiliations:** 1 Key Laboratory of Tropical Plant Resource and Sustainable Use, Xishuangbanna Tropical Botanical Garden, Chinese Academy of Sciences, Kunming 650223, China; 2 Faculty of Life Science, University of Chinese Academy of Sciences, Beijing 100049, China

**Keywords:** *Tectona grandis*, chemical composition, antioxidant activity, anti-inflammatory activity, *α*-glucosidase, glucose uptake

## Abstract

*Tectona grandis* is a traditional Dai medicine plant belonging to the Lamiaceae family, which can be used to treat malaria, inflammation, diabetes, liver disease, bronchitis, tumors, cholelithiasis, jaundice, skin disease and as an anti-helminthic. To find more novel therapeutic agents contained in this medicinal plant, the antioxidant, anti-inflammatory and anti-diabetic activities of *T. grandis* methanolic extract, fractions and compounds were evaluated. In this study, 26 compounds were isolated from the leaves and branches of *T. grandis*. Their structures were identified based on extensive spectral experiments, including NMR, ESI-MS and comparison with published spectral data. Among them, compounds **1**–**2**, **4**–**6**, **9**–**14** and **16**–**22** were reported for the first time for this plant. The antioxidant activity screening results showed that compounds **5**, **15** and **23** had potent antioxidant capacities, with SC_50_ values from 0.32 to 9.92 µmol/L, 0.92 to 1.10 mmol Trolox/L and 1.02 to 1.22 mmol Trolox/L for DPPH, ABTS and FRAP, respectively. In addition, their anti-inflammatory effects were investigated by releasing TNF-*α*, IL-1*β* and IL-6 through the use of mouse monocytic macrophages (RAW 264.7). Compounds **1**, **13**, **18** and **23** had the effects of reducing the expression of inflammatory factors. Compounds **13** and **18** were reported for the first time for their anti-inflammatory activities. Furthermore, the methanolic extract (ME), petroleum ether extract (PEE) and EtOAc extract (EAE) of *T. grandis* showed significant glucose uptake activities; compounds **21** and **23** significantly promoted glucose uptake of 3T3-L1 adipocytes at 40 µM. Meanwhile, compounds **4**, **5** and **7** showed significant inhibitory activities against α-glucosidase, with IC_50_ values of 14.16 ± 0.34 µmol/L, 19.29 ± 0.26 µmol/L and 3.04 ± 0.08 µmol/L, respectively. Compounds **4** and **5** were reported for the first time for their *α*-glucosidase inhibitory activities. Our investigation explored the possible therapeutic material basis of *T. grandis* to prevent oxidative stress and related diseases, especially inflammation and diabetes.

## 1. Introduction

*Tectona grandis* has been widely used in traditional Dai medicine. It mainly grows in tropical and subtropical southwestern China, India, Laos and northern Thailand. It is a large deciduous tree, measuring up to 40–50 m, with a deeply fluted trunk that can reach 2–2.5 m in diameter and a brown or gray bark [1]. Previous phytochemical research reported that *T. grandis* was not only rich in flavonoids and quinones, but also contained phenolic, steroids, phenylpropanoids, fatty esters and other compounds [2]. In ethnomedicine, *T. grandis* is commonly used to treat wounds, pain, fever, malaria, inflammation, diabetes, liver disease, helminthic infection, bronchitis, tumors, cholelithiasis, jaundice, skin disease and bacterial infection [3,4,5,6,7]. Pharmacological studies conducted on the methanolic extracts of *T. grandis* bark and flowers established its hypoglycemic activities [8,9]. The leaf extract of *T. grandis* has significant wound healing activity [10]. Hydrochloric acid extract of *T. grandis* leaves exhibited antitumor activity in the female Swiss mouse malaria model, thereby validating its traditional use in the treatment of tumors [11]. Traditional use of *T. grandis* as an antibacterial and anti-inflammatory medicine was validated by a study conducted by Bitchagno [12], who reported that the ethanolic extract from the fruit of *T. grandis* exhibited a remarkable inhibitory effect on four Gram-negative bacteria, and the methanolic extract of *T. grandis* woods demonstrated significant analgesic activity and inhibited edema action in writhing test and paw edema test rats [13]. In summary, *T. grandis* has a wide range of pharmacological properties. However, the previous modern pharmacological research on *T. grandis* mainly focused on antibacterial and analgesic aspects [14,15]; the components of antioxidative, anti-inflammatory and anti-diabetes effects of *T. grandis* have not yet been studied in detail. It is worthwhile to explore the therapeutic material basis and effects of *T. grandis* in treating oxidative stress and related diseases, such as inflammation and diabetes.

Reactive oxygen species (ROS) have important roles in a wide range of physiological processes; however, oxidative stress and the resultant oxidative damage have been implicated in many human diseases, including cardiovascular disease, neurodegenerative diseases, inflammation, diabetes and cancer and also in the aging process [16,17,18]. Extensive or prolonged exposure to ROS results in oxidative stress, which is a deleterious process that damages lipids, proteins and DNA in the cell. Excessive ROS can cause damage to cell structure and function and induce somatic cell mutation and tumor transformation [19]. It is noteworthy that excessive ROS produced during oxidative metabolism can induce inflammatory processes leading to the production of many inflammatory mediators such as TNF-*α*, IL-1*β* and IL-6 [20], which can lead to a variety of chronic diseases. As such, antioxidants play an important role in anti-inflammation and protection against oxidative damage to proteins and DNA. Meanwhile, oxidative stress and inflammation play a key role in the development of diabetes and its complications [21,22]. When excessive amounts of ROS are produced in the body, the internal oxidation and antioxidant effects will be out of balance, and lead to oxidative stress and ultimately damage the macromolecules involved in insulin release [23]. According to the International Diabetes Federation (IDF), globally, the number of cases of diabetes are predicted to increase from 537 million in 2021 to 643 million by 2030 [24]. *α*-Glucosidase is an important catalytic enzyme involved in the hydrolysis of carbohydrates in the gastrointestinal tract, which enables monosaccharides to be absorbed into the blood, thus reducing postprandial glucose fluctuations in diabetic patients. It has been confirmed that *α*-glucosidase inhibitors possessing delayed *α*-glucose uptake can reduce postprandial blood glucose levels [25]. In view of this, one of the therapeutic strategies used to manage diabetes focuses on the inhibition of *α*-glucosidase.

Herein, we reported the isolation and structure identification of compounds **1**–**26** from *T. grandis*, together with the exploration of their potential antioxidant, anti-inflammatory, *α*-glucosidase inhibition and glucose uptake activities. Firstly, DPPH, ABTS and FRAP methods were used to detect and analyze the antioxidant activities and the protective effects against oxidative damage to DNA and protein. Secondly, different types of compounds were screened for the inhibitory effects of TNF-*α*, IL-1*β* and IL-6 inflammatory factors. Finally, glucose uptake assays and *α*-glucosidase inhibition experiments were used to explore its anti-diabetes effect. The potent antioxidant capacities of compounds **15** and **23** suggested that they might be potential natural candidate drugs to inhibit oxidative stress and prevent DNA and protein oxidative damage.

## 2. Materials and Methods

### 2.1. Plant Material

Branches and leaves of *T. grandis* were collected from Xishuangbanna Tropical Botanical Garden, Chinese Academy of Sciences, in August 2020. The original plants were identified by Hua Shuai, an engineer at the Xishuangbanna Tropical Botanical Garden, Chinese Academy of Sciences. The voucher specimen (20200801) was given to the Innovative Drug Research Group, Xishuangbanna Tropical Botanical Garden, Chinese Academy of Sciences.

### 2.2. General Experimental Procedures

ESI-MS was carried out using a Bruker Micro ToF-Q II mass spectrometer (Bruker Daltonics, Fremont, CA, USA). NMR spectra were recorded using a Bruker AV II-600 or 400 MHz spectrometer (Bruker, Fällanden, Switzerland). Column chromatography (CC) was run on silica gel (80–100 mesh or 200–300 mesh) (Qingdao Marine Chemical Co., Ltd., Qingdao, China), LiChroprep RP-C18 gel (Merck, 40–63 μm) and Sephadex LH-20 (GE Healthcare). Fractions were monitored using thin layer chromatography (TLC) and spots were visualized by heating silica gel plates sprayed with 10% H_2_SO_4_/CH_3_CH_2_OH. Semipreparative HPLC was run on a Shimadzu system (Shimadzu Corporation, Nakagyo-ku, Kyoto, Japan) with a Shim-pack Scepter C18-120 (4.6 mm × 250 mm, 5 µm). RAW 264.7 mouse mononuclear macrophages and 3T3-L1 mouse preadipocytes were purchased from the American Type Culture Collection (ATCC, Manassas, VA, USA). High glucose DMEM, low glucose DMEM, Pen-Strep solution (P/S), insulin, certified fetal bovine serum (FBS), special newborn calf serum (NBCS) and phosphate-buffered saline (PBS) were purchased from Biological Industries (Shanghai, China). 3-Isobutyl-1-methylxanthine (IBMX) and dexamethasone (DEX) were obtained from Sigma-Aldrich (St. Louis, MO, USA). Rosiglitazone (ROSI) was purchased from Meilun Biotech Co., Ltd. (Dalian, Liaoning, China). Ascorbic acid, folin-phenol, 5 × protein loading buffer, the sodium dodecyl sulfate-polyacrylamide gel electrophoresis (SDS-PAGE) gel kit and dimethyl sulfoxide (DMSO) was obtained from Solarbio (Beijing, China). Lipopolysaccharide (LPS) was obtained from ACMEC (Beijing, China). The cytokine kit was purchased from Kunming Zanna Biotech Co., Ltd. (Kunming, China), glucose test kit was purchased from Rongsheng Biotech Co., Ltd. (Shanghai, China). CellTiter 96^®^ AQueous One Solution Cell Proliferation Assay was obtained from Promega Corporation (Madison, WI, USA). *α*-Glucosidase (33 U/mg), acarbose, 4-nitrophenyl-*α*-D-glucopyranoside (*p*NPG) and ascorbic acid were purchased from Yuanye Biotech Co., Ltd. (Shanghai, China). The absorbance was measured using a microplate reader (Molecular Devices, Palo Alto, Santa Clara, CA, USA). 2,2′-Azobis (2-methylpropionamidine) dihydrochloride (AAPH) was purchased from GlpBio (Montclair, America). pBR322 DNA was purchased from Takara (Beijing, China). The 5 × DNA loading buffer was purchased from Shanghai Generay Biotech Co., Ltd. (Shanghai, China). Agarose and rutin were obtained from Aladdin (Shanghai, China). Bovine serum albumin (BSA) was purchased from BioFroxx (Einhausen, Germany). 2,2′-azino-bis (3-ethylbenzothiazoline-6-sulfonic acid) (ABTS) and ferric-reducing antioxidant power (FRAP) detection reagents were purchased from Suzhou Comin Biotechnology Co., Ltd. (Jiangsu, China). Gallic acid, sodium nitrite, aluminum nitrate, sodium carbonate, 2,2-diphenyl-1-picrylhydrazyl (DPPH), brilliant blue R and sodium hydroxide were purchased from Macklin (Shanghai, China). The other chemicals and reagents were purchased from local suppliers.

### 2.3. Extraction, Isolation and Purification

Dried branches and leaves of *T. grandis* (16 kg) were extracted using heating reflux for 3 h in 90% methanolic (100 L × 3 times). The filtrate was combined and concentrated under vacuum to obtain a methanolic extract (ME, 1.55 kg), then sequentially partitioned with petroleum ether, EtOAc, n-BuOH and water, respectively, to obtain petroleum ether extract (PEE, 793 g), EtOAc extract (EAE, 141 g), n-BuOH extract (NBE, 247 g) and water extract (WE, 369 g). 

The EtOAc extract (141 g) was purified using silica gel CC and eluted with a gradient of petroleum ether: EtOAc (from 50:1 to 1:1, *v*/*v*) and CHCl_3_: MeOH (from 10:1 to 1:1, *v*/*v*) to provide six fractions (Fr. A–Fr. F). Fr. C (15.9 g) was further isolated using silica gel CC eluted with petroleum ether: EtOAc (from 100:1 to 1:1, *v*/*v*) to obtain three subfractions (C1–C3). Subfraction C3 (9.23 g) was further isolated using silica gel CC eluted with petroleum ether: EtOAc (from 50:1 to 1:1, *v*/*v*), Sephadex LH-20 eluted with CHCl_3_: MeOH (1:1, *v*/*v*), semi-preparative HPLC and eluted with methanol: H_2_O (45:55, *v*/*v*) to obtain compounds **9** (1.2 mg), **10** (2 mg), **11** (15.8 mg) and **12** (2.8 mg). Fr. D (95.4 g) was purified repeatedly using silica gel CC eluted with EtOAc: MeOH (from 80:0 to 0:1, *v*/*v*) to obtain four subfractions (D1–D4). Subfraction D2 (39.23 g) was purified repeatedly using silica gel CC eluted with EtOAc: MeOH (from 50:0 to 0:1, *v*/*v*), MCI (small pore resin gel column, polystyrene-based inverse resin filler) eluted with MeOH: H_2_O (from 10:90 to 100:0, *v*/*v*), Sephadex LH-20 eluted with CHCl_3_: MeOH (from 3:1 to 1:1, *v*/*v*), semi-preparative HPLC eluted with methanol: H_2_O (form 70:30 to 55:45, *v*/*v*) to obtain compounds **14** (2 mg), **16** (4 mg), **17** (10 mg), **18** (1.9 mg), **22** (0.8 mg) and **23** (4.9 mg). Fr. E (25 g) was chromatographed repeatedly with a gradient of petroleum ether: EtOAc (from 100:0 to 1:1, *v*/*v*), ODS (octadecylsilane) and eluted with petroleum ether: EtOAc (from 10:1 to 1:1, *v*/*v*), silica gel CC eluted with petroleum ether: EtOAc (from 50:1 to 1:1, *v*/*v*), Sephadex LH-20 eluted with CHCl_3_: MeOH (1:1, *v*/*v*) to obtain compounds **13** (4 mg), **19** (3.7 mg), **20** (2.4 mg) and **21** (5 mg). 

The n-BuOH extract (247.3 g) was purified using D101 macroporous resin and eluted with EtOH: H_2_O (from 20:80 to 100:0, *v*/*v*) to obtain three fractions (Fr. I–Fr. III). Fr. I (66.3 g) was purified repeatedly using silica gel CC eluted with CHCl_3_: MeOH (from 100:0 to 1:1, *v*/*v*) to obtain four subfractions (I1–I4). Subfraction I2 (15.7 g) was purified repeatedly using silica gel CC eluted with CHCl_3_: MeOH (from 20:0 to 1:1, *v*/*v*), MCI eluted with MeOH: H_2_O (from 10:90 to 100:0, *v*/*v*), Sephadex LH-20 eluted with CHCl_3_: MeOH (1:1, *v*/*v*), semi-preparative HPLC eluted with methanol: H_2_O (from 70:30 to 50:50, *v*/*v*) to obtain compounds **1** (2.1 mg), **2** (0.8 mg), **3** (1.2 mg), **4** (1.6 mg) and **5** (1 mg). Fr. II (25.9 g) was further isolated using silica gel CC eluted with petroleum ether: EtOAc (from 100:1 to 10:1, *v*/*v*) to obtain compounds **15** (236 mg), **24** (200.7 mg), **25** (209 mg) and **26** (50.7 mg). Fr. III (35.2 g) was purified repeatedly using silica gel CC eluted with CHCl_3_: MeOH (from 100:1 to 1:1, *v*/*v*) and petroleum ether: EtOAc (from 80:1 to 1:1, *v*/*v*), Sephadex LH-20 eluted with CHCl_3_: MeOH (1:1, *v*/*v*), semi-preparative HPLC eluted with methanol: H_2_O (80:20, *v*/*v*) to obtain compounds **6** (30.6 mg), **7** (8 mg) and **8** (0.6 mg).

### 2.4. DPPH Free Radical Scavenging Assay

The DPPH free radical scavenging activities of *T. grandis* methanolic extract, different fractions and its compounds were conducted according to Dr. Yang et al. [26]. The concentration gradient of methanolic extract and different fractions was 160.0, 80.0, 40.0, 20.0, 10.0, 5.0 and 2.5 μg/mL, the concentration gradient of VC was 100.0, 50.0, 25.0, 12.5, 6.3, 3.1 and 1.6 μg/mL and the detection concentration of compounds (**1**–**23**) was 50 µM. Initially, the samples were dissolved in DMSO to different concentrations, and the DPPH was dissolved in PBS (0.1 M, pH 6.8) to 0.1 mM. Then, 190 µL DPPH solution and 10 µL sample were mixed in each well of a 96-well plate and incubated for 30 min at room temperature in the dark. The absorbance was measured using a microplate reader at 517 nm, and ascorbic acid was used as a positive control. The DPPH scavenging activity was calculated according to the following formula:

Scavenging activity (%) = [1 − (A1 − A2)/(A3 − A4)] × 100% (1)

where A1 is the OD value of the tested samples, A2 is the OD value of the sample control, A3 is the OD value of the negative control and A4 is the OD value of the blank control. The analysis was performed in triplicates and the results were described as SC_50_ values.

### 2.5. ABTS Radical Cation Scavenging Assay

The ABTS radical cation (ABTS•+) scavenging activities were conducted in accordance with the instructions for the ABTS kit. The total antioxidant activity values were estimated using the Trolox equivalent antioxidant capacity test. The detection concentration of methanolic extract and different fractions was 80 μg/mL, and the detection concentration of compounds (**1**–**23**) was 50 μM. The ABTS working solution was prepared by taking 1 bottle of reagent 2 and adding 11 mL reagent 1, shaking it for 20 min, letting it stand and taking the supernatant for use. Then, 190 μL of ABTS working reagent was mixed with 10 μL sample and incubated at room temperature for 5 min. The absorbance was then measured using a microplate reader at 734 nm, and the scavenging activity was calculated according to Equation (2). The blank control group used DMSO to replace the sample.

Scavenging activity (mmol Trolox/L) = 1.424 × (A1 − A2 + 0.0012)(2)

where A1 is the OD value of the blank control and A2 is the OD value of the tested samples. The analysis was performed in triplicates, 1.424, 0.0012 was a constant.

### 2.6. Ferric Reducing Antioxidant Power Assay

The FRAP assay was determined in accordance with the instructions for the FRAP kit. The total antioxidant activity values were estimated using the Trolox equivalent antioxidant capacity test. Initially, the detection concentration of methanolic extract and different fractions was 80 μg/mL, and the detection concentration of compounds (**1**–**23**) was 50 μM. Then, 190 μL of FRAP working reagent was mixed with 10 μL sample and incubated at room temperature for 20 min. The absorbance was then measured using a microplate reader at 593 nm, and the scavenging activity was calculated according to Equation (3). The blank control group used DMSO to replace the sample.

Scavenging activity (mmol Trolox/L) = 0.8054 × (A1 − A2 − 0.0134)(3)

where A1 is the OD value of the blank control and A2 is the OD value of the tested samples. The analysis was performed in triplicates, 0.8054, 0.0134 was a constant.

### 2.7. DNA Oxidative Damage Assay

The effects of *T. grandis* methanolic extract, different fractions and its compounds **15**, **19** and **23** on the prevention of DNA oxidative damage were carried out using the method of Han et al. [27]. Initially, 8 μL pBR322 DNA (50 μg/mL) in PBS (0.1 M, pH 6.8) was mixed with different concentrations of *T. grandis* fractions and its compounds (12 μL) and incubated in water at 37 °C for 30 min. Then, 5 μL AAPH (20 mM) was added to the mixture and the reaction continued for 60 min. The control group used the same volume of PBS (0.1 M, pH 6.8) to replace AAPH. The pre-treated samples were mixed with 5 × DNA loading buffer, and the mixture (8 μL) was taken for 1.0% agarose gel electrophoresis for 25 min. After electrophoresis, the gels were stained with ethidium bromide for 20 min. Finally, images were taken using a DNA gel imaging system (ChampChemi 610, Beijing sage creation Co., Ltd. Beijing, China). 

### 2.8. Protein Oxidative Damage Assay

The protective activities of *T. grandis* methanolic extract, different fractions and its compounds **15** and **23** on protein oxidative damage were carried out using the method of Yang et al. [28]. Initially, 20 μL BSA solutions (1 mg/mL) in PBS (0.1 M, pH 6.8) were mixed with different concentrations of fractions (40 μL) and compounds (40 μL) and incubated in water at 37 °C for 30 min. Then, 40 μL AAPH (160 mM) was added to the mixture and the reaction continued for 4 h. The control group used the same volume of PBS (0.1 M, pH 6.8) to replace AAPH. The pre-treated samples were added to 5 × SDS loading buffer and treated in water at 95 °C for 15 min. The mixture (16 μL) was taken for 10% SDS-PAGE for 95 min. After electrophoresis, the gels were stained with Coomassie brilliant blue R-250 dye (0.25%, *w*/*v*), and then the gels were decolorized with the decolorizing solution. Finally, images were taken using the SDS-PAGE gel imaging system (ChampChemi 610, China) and the grayscale of Western blot was analyzed using Image J software.

### 2.9. Anti-Inflammatory Activity and Cell Viability Assay

In order to increase cytokine production, macrophages were treated with LPS at a final concentration of 1 µg/mL, and then with *T. grandis* compounds at 40 µM. The compounds were solubilized in DMEM medium containing 0.1% DMSO. Dexamethasone was employed as a positive control (5 µg/mL). A centrifuge was used to separate the supernatant from the cells after 48 h of incubation, with three replicates per group. Quantification of TNF-*α*, IL-1*β* and IL-6 secretion was achieved by following the ELISA manufacturer's instructions. Then, absorbance was measured at 450 nm. Relative TNF-*α*, IL-1*β* and IL-6 expression was calculated according to Equation (4). Following the inflammatory factor test, cell viability was detected by CellTiter96^®^ Aqueous One Solution Cell Proliferation Assay. Then, 20 μL/well CellTiter96^®^ Aqueous One Solution Cell Growth Assay reagent was added to the plate and incubated at 37 °C for 8 h, absorbance was measured at 570 nm and relative cell viability was calculated according to Equation (5).

Relative content of inflammatory cytokines (%) = (A1/A2)/A3 × 100%(4)

where A1 is the concentrations value of inflammatory cytokines of each group, A2 is the relative cell viability of each group (model group is 100) and A3 is the concentration of inflammatory cytokines of model group.

Cell viability rate (%) = B1/B2 × 100%(5)

where B1 is the OD value of each fraction group or positive control group and B2 is the OD value of the model control.

### 2.10. α-Glucosidase Inhibition Assay

The inhibitory effect on *α*-glucosidase was measured according to the previous report method with slight modifications [29]. The 20 mM sample dissolved in DMSO was diluted to 50 μM in PBS, 10 μL sample was mixed with 50 μL of 0.1 μ/mL *α*-glucosidase solution in 0.1 M phosphate buffer (pH 6.5) in a 96-well microplate and 10 μL PBS was used as a blank control. The mixture was incubated at 37 °C for 10 min before adding 40 µL of 5 mM 4-nitrophenyl-*α*-D-glucopyranoside to each well. After 30 min of incubation at 37 °C, the reaction was terminated by adding 50 μL of 0.1 M Na_2_CO_3_ to this mixture. The released 4-nitrophenol absorbance measurements were carried out using a microplate reader (SpectraMax190, USA) at 405 nm. Acarbose was used as the positive control. The enzyme inhibitory activity was expressed as % inhibition and was calculated via the equation:

Inhibition rate (%) = (A1 − A2)/A1 × 100%(6)

where A1 is the OD value of the negative control and A2 is the OD value of each fraction group or positive control group.

### 2.11. Glucose Uptake and Cell Viability Assay

Mouse 3T3-L1 preadipocyte cells were maintained in DMEM supplemented with 10% NBCS and 1% P/S at 37 °C in a humidified environment with 5% CO_2_, and then began starvation when the cells grew to 80% confluence (day 0). To facilitate differentiation to adipocytes, 2-days post-confluent cells were placed in 10% FBS DMEM supplemented with 1% P/S, 0.5 mM IBMX, 1 μM Dex, 1 μM Rosi and 100 nM insulin (day 2). For another three days (day 5), the medium was changed to high glucose DMEM containing 10% FBS, 1% P/S and 100 nM insulin for one day (day 6), and the 3T3-L1 preadipocytes were fully differentiated into mature adipocytes. 

Next, differentiated 3T3-L1 adipocytes at a density of 5 × 10^4^ cells/well were cultured with DMEM in 96-well plates and divided into the blank control group, model group, insulin group (100 ng/mL), berberine group (10 μg/mL) and compound groups (40 μM). Then, 10 μL samples were mixed with 190 μL medium and added to individual 3T3-L1 adipocytes. This was repeated 3 times. After 24 and 48 h of culture, glucose uptake was initiated by the addition of 10 μM medium and 190 μL glucose detection reagent to each well, reaction at 37 °C for 15 min, and the glucose uptake was measured according to the operating instructions of the glucose content determination kit. Following the glucose uptake test, cell viability was detected by CellTiter96^®^ Aqueous One Solution Cell Proliferation Assay. Then, 20 μL/well CellTiter96^®^ Aqueous One Solution Cell Growth Assay reagent was added to the plate and incubated at 37 °C for 8 h. Absorbance was measured at 490 nm and relative cell viability was calculated according to Equation (7).

Cell viability rate (%) = A1/A2 × 100% (7)

where A1 is the OD value of each fraction group or positive control group and A2 is the OD value of the blank control.

### 2.12. Statistical Analysis

All data are presented as the means ± SD from 3 replicates. The differences between different samples were assessed using a one-way analysis of variance (ANOVA). It was considered a significant difference when the *p* value was less than 0.05. All analyses were performed using Graph Pad Prism 7.0 software (Graph Pad Software Inc., San Diego, CA, USA).

## 3. Results and Discussion

### 3.1. Structure Elucidation 

**Figure 1:**
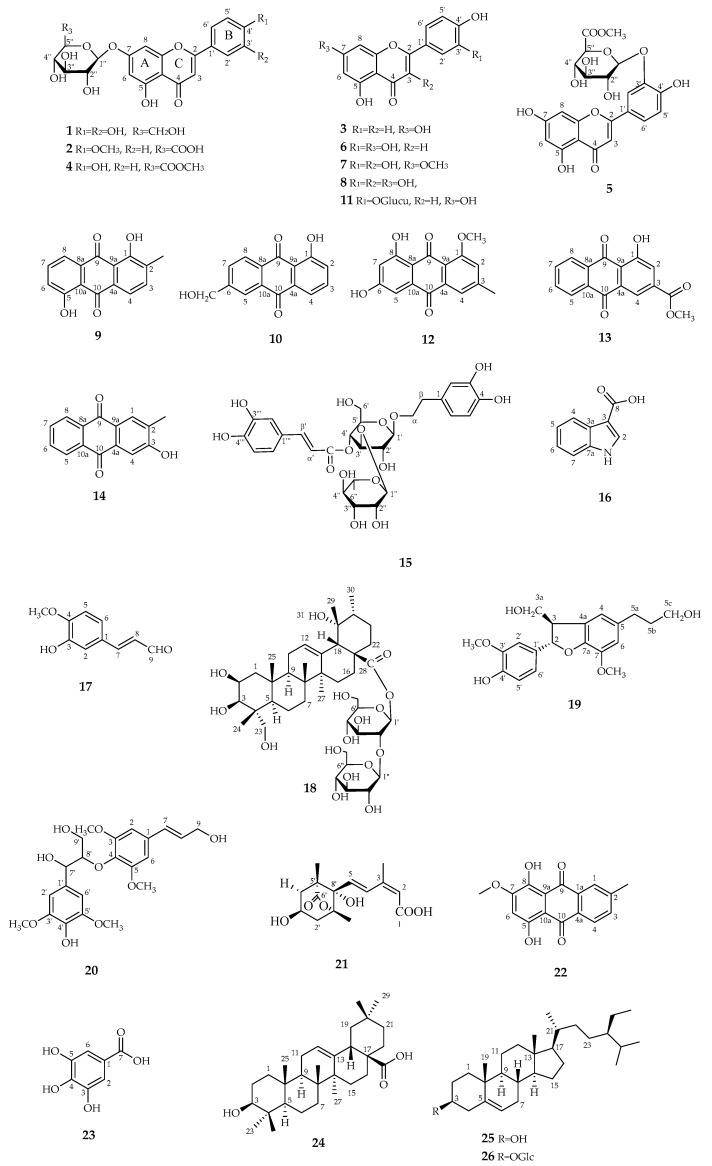
Structures of compounds **1**–**26** from *T. grandis*.

Twenty-six compounds (Figure 1) were isolated from the dried branches and leaves of *T. grandis*, including luteolin-7-*O*-*β*-D-glucoside (**1**) [30], acacetin-7-*O*-*β*-glucuronide (**2**) [31], apigenin (**3**) [32], apigenin-7-*O*-*β*-D-glucuronide methyl ester (**4**) [33], vitegnoside (**5**) [34], luteolin (**6**) [35], rhamnetin (**7**) [36], quercetin (**8**) [37], isozyganein (**9**) [38], 1-hydroxy-6-hydroxymethyl anthraquinone (**10**) [39], luteolin-3′-*O*-glucuronide (**11**) [40], 1-*O*-methylemodin (**12**) [41], 3-carbomethoxy-1-hydroxy-9,10-anthraquinone (**13**) [42], 3-hydroxy-2-methyl-9,10-anthraquinone (**14**) [43], verbascoside (**15**) [44], 1H-indole-3-carboxylic acid (**16**) [45], 3-hydroxy-4-methoxycinnamaladehyde (**17**) [46], 2*β*,3*β*,19*α*,23-tetrahydroxy-urs-12-en-28-*O*-[*β*-D-glucopyranosyl (1-2)-*β*-D-glucopyranosyl] ester (**18**) [47], rel-(2*α*,3*β*)-7-*O*-methylcedrusin (**19**) [48], 7*S*,8*R*-syringylglycerol-8-*O*-4′-(synapyl alcohol) ether (**20**) [49], rel-5-(3*S*,8*S*-dihydroxy-1*R*,5*S*-dimethyl-7-oxa-6-oxobicyclo [1–3]oct-8-yl)-3-methyl-2*Z*,4*E*-pentadienoic acid (**21**) [50], austrocortirubin (**22**) [51], gallic acid (**23**) [52], oleanolic acid (**24**) [53], *β*-sitosterol (**25**) [54] and *β*-sitosterol 3-*O*-*β*-D-glucopyranoside (**26**) [55]. Among them, compounds **1**–**2**, **4**–**6**, **9**–**14** and **16**–**22** were isolated from *T. grandis* for the first time.

**Compound 1.** C_21_H_20_O_11_. ESI-MS *m*/*z* 447 [M-H]^−^; ^1^H NMR (CD_3_OD, 500 MHz,) *δ*_H_ 7.79 (1H, d, H-2′), 7.62 (1H, dd, *J* = 8.5, 2.0 Hz, H-6′), 6.99 (1H, d, *J* = 8.5 Hz, H-5′), 6.60 (1H, s, H-3), 6.46 (1H, d, H-8), 6.21 (1H, d, *J* = 2.0 Hz, H-6), 4.61 (1H, s, H-1″), 4.15 (1H, d, *J* = 12.0 Hz, H-6″a), 3.84 (1H, dd, *J* = 12.0, 6.6 Hz, H-6″b), 3.64 (1H, m, H-5″), 3.58 (2H, overlapped, H-2″,3″), 3.44 (1H, m, H-4″); ^13^C NMR (CD_3_OD, 125 MHz) *δ*_C_ 183.9 (C-4), 166.5 (C-7), 165.6 (C-2), 163.3 (C-9), 159.5 (C-5), 153.2 (C-4′), 147.0 (C-3′), 124.0 (C-1′), 123.6 (C-6′), 118.2 (C-5′), 116.9 (C-2′), 105.3 (C-10), 104.3 (C-3), 104.24 (C-1″), 100.3 (C-6), 95.3 (C-8), 76.9 (C-2″), 76.8 (C-3″), 74.6 (C-4″), 72.9 (C-5″), 64.4 (C-6″).

**Compound 2.** C_22_H_20_O_11_. ESI-MS *m*/*z* 459 [M-H]^−^; ^1^H NMR (DMSO-*d_6_*, 500 MHz) *δ*_H_ 12.98 (1H, s, 5-OH), 7.96 (2H, d, *J* = 8.8 Hz, H-2′,6′), 6.94 (2H, d, *J* = 8.8 Hz, H-3′,5′), 6.87 (1H, s, H-3), 6.86 (1H, d, *J* = 2.2 Hz, H-8), 6.47 (1H, d, *J* = 2.2 Hz, H-6), 5.36 (1H, s, H-1″), 4.20 (1H, d, *J* = 9.6 Hz, H-5″), 3.66 (3H, s, 4′-OCH_3_), 3.41–3.34 (2H, overlapped, H-3″,4″), 3.31 (1H, m, H-2″); ^13^C NMR (DMSO-*d_6_*, 125 MHz,) *δ*_C_ 196.5 (C-6″), 182.0 (C-4), 164.3 (C-7), 162.4 (C-2), 161.4 (C-5), 161.2 (C-4′), 156.9 (C-9), 128.6 (C-2′), 128.6 (C-6′), 121.0 (C-1′), 116.0 (C-3′), 116.0 (C-5′), 105.5 (C-10), 103.1 (C-3), 99.3 (C-1″), 99.0 (C-6), 94.6 (C-8), 75.4 (C-3″), 75.1 (C-5″), 72.7 (C-2″), 71.3 (C-4″), 48.6 (4′-OCH_3_).

**Compound 3.** C_15_H_10_O_5_. ESI-MS *m*/*z* 269 [M-H]^−^; ^1^H NMR (DMSO-*d_6_*, 600 MHz) *δ*_H_ 12.90 (1H, s, 5-OH), 7.82 (2H, d, *J* = 8.7 Hz, H-2′,6′), 6.90 (2H, d, *J* = 8.7 Hz, H-3′,5′), 6.52 (1H, d, *J* = 2.2 Hz, H-8), 6.51 (1H, s, H-3), 6.42 (1H, d, *J* = 2.2 Hz, H-6); ^13^C NMR (DMSO-*d_6_*, 150 MHz) *δ*_C_ 183.1 (C-4), 165.1 (C-7), 164.9 (C-2), 163.4 (C-5), 161.9 (C-4′), 158.8 (C-9), 129.3 (C-2′,6′), 123.3 (C-1′), 116.9 (C-3′,5′), 105.4 (C-10), 104.1 (C-3), 99.7 (C-6), 94.7 (C-8).

**Compound 4.** C_22_H_20_O_11_. ESI-MS *m*/*z* 459 [M-H]^−^; ^1^H NMR (DMSO-*d_6_*, 600 MHz) *δ*_H_ 7.95 (2H, d, *J* = 8.8 Hz, H-2′,6′), 6.94 (2H, d, *J* = 8.8 Hz, H-3′,5′), 6.86 (2H, d, *J* = 2.1 Hz, H-3,8), 6.47 (1H, d, *J* = 2.1 Hz, H-6), 5.31 (1H, d, *J* = 7.4 Hz, H-1″), 3.66 (3H, s, H-5″); ^13^C NMR (DMSO-*d_6_*, 150 MHz) *δ*_C_ 182.0 (C-4), 169.3 (C-6″), 164.4 (C-2), 162.4 (C-7), 161.5 (C-5), 161.2 (C-4′), 157.0 (C-9), 128.7 (C-2′), 128.7 (C-6′), 120.9 (C-1′), 116.1 (C-3′,5′), 105.5 (C-10), 103.1 (C-3), 99.3 (C-6), 99.0 (C-1″), 94.7 (C-8), 75.4 (C-3″), 75.2 (C-5″), 72.7 (C-2″), 71.3 (C-4″), 52.1 (5″-OCH_3_).

**Compound 5.** C_22_H_20_O_12_. ESI-MS *m*/*z* 475 [M-H]^−^; ^1^H NMR (DMSO-*d_6_*, 600 MHz) *δ*_H_ 12.93 (1H, s, 5-OH), 7.68–7.61 (2H, m, H-2′,6′), 6.96 (1H, d, *J* = 8.4 Hz, H-5′), 6.81 (1H, s, H-3), 6.46 (1H, d, *J* = 1.8 Hz, H-8), 6.19 (1H, d, *J* = 1.8 Hz, H-6), 5.22 (1H, d, *J* = 7.3 Hz, H-1″), 4.21 (1H, d, *J* = 9.7 Hz, H-5″), 3.30–3.38 (3H, overlapped, H-3″,2″,4″), 3.16 (3H, s, 6″-OCH_3_); ^13^C NMR (DMSO-*d_6_*, 150 MHz) *δ*_C_ 181.7 (C-4), 169.3 (C-6″), 164.5 (C-7), 163.3 (C-2), 161.4 (C-5), 157.3 (C-9), 151.3 (C-4′), 145.2 (C-5′), 122.0 (C-2′), 121.1 (C-1′), 116.8 (C-3′), 113.9 (C-6′), 103.6 (C-10), 103.1 (C-3), 100.8 (C-1″), 98.9 (C-6), 94.0 (C-8), 75.2 (C-5″), 75.1 (C-3″), 72.9 (C-2″), 71.4 (C-4″), 52.1 (5″-OCH_3_).

**Compound 6.** C_15_H_10_O_6_. ESI-MS *m*/*z* 285 [M-H]^−^; ^1^H NMR (DMSO-*d_6_*, 500 MHz) *δ*_H_ 7.41 (1H, d, *J* = 2.1 Hz, H-6′), 7.39 (1H, d, *J* = 2.1 Hz, H-2′), 6.88 (1H, 1H, H-5′), 6.65 (1H, 1H, H-3), 6.42 (1H, s, *J* = 1.4 Hz, H-8), 6.17 (1H, s, *J* = 1.4 Hz, H-6); ^13^C NMR (DMSO-*d_6_*, 125 MHz) *δ*_C_ 181.6 (C-4), 164.2 (C-2), 163.8 (C-7), 161.4 (C-9), 157.3 (C-5), 149.7 (C-4′), 145.7 (C-3′), 121.4 (C-1′), 118.9 (C-6′), 116.0 (C-5′), 113.3 (C-2′), 103.6 (C-10), 102.8 (C-3), 98.8 (C-6), 93.8 (C-8).

**Compound 7.** C_16_H_12_O_7_. ESI-MS *m*/*z* 315 [M-H]^−^; ^1^H NMR (DMSO-*d_6_*, 500 MHz) *δ*_H_ 12.48 (1H, s, 5-OH), 7.71 (1H, d, *J* = 2.1 Hz, H-2′), 7.56 (1H, dd, *J* = 8.5, 2.1 Hz, H-6′), 6.88 (1H, d, *J* = 8.5 Hz, H-5′), 6.69 (1H, d, *J* = 2.1 Hz, H-8), 6.34 (1H, d, *J* = 2.1 Hz, H-6), 3.85 (3H, s, 7-OCH_3_); ^13^C NMR (DMSO-*d_6_*, 125 MHz) *δ*_C_ 175.9 (C-4), 164.8 (C-7), 160.3 (C-5), 156.0 (C-9), 147.8 (C-2), 147.2 (C-4′), 145.0 (C-3′), 136.0 (C-3), 121.8 (C-6′), 120.0 (C-1′), 115.5 (C-2′), 115.2 (C-5′), 104.0 (C-10), 97.4 (C-6), 91.9 (C-8), 56.0 (7-OCH_3_).

**Compound 8.** C_15_H_10_O_7_. ESI-MS *m*/*z* 301 [M-H]^−^; ^1^H NMR (DMSO-*d_6_*, 500 MHz) *δ*_H_ 7.74 (1H, s, H-2′), 7.64 (1H, d, *J* = 8.4, 2.0 Hz, H-6′), 6.88 (1H, d, *J* = 8.4 Hz, H-5′), 6.39 (1H, d, *J* = 2.0 Hz, H-8), 6.19–6.17 (1H, m, *J* = 2.0 Hz, H-6); ^13^C NMR (DMSO-*d_6_*, 125 MHz) *δ*_C_ 177.4 (C-4), 165.7 (C-7), 162.6 (C-5), 158.3 (C-9), 148.9 (C-2), 148.1 (C-4′), 146.3 (C-3′), 137.3 (C-3), 124.2(C-1′), 121.7 (C-6′), 116.3 (C-5′), 116.1 (C-2′), 104.6 (C-10), 99.3 (C-6), 94.5 (C-8).

**Compound 9.** C_15_H_10_O_4_. ESI-MS *m*/*z* 253 [M-H]^−^; ^1^H NMR (CD_3_OD, 600 MHz) *δ*_H_ 8.04 (1H, s, *J* = 8.4 Hz, H-8), 7.72 (1H, d, *J* = 8.4 Hz, H-4), 7.67 (1H, t, *J* = 7.8 Hz, H-7), 7.52 (1H, s, H-3), 7.26 (1H, d, *J* = 7.8 Hz, H-6), 2.33 (3H, s, 2-CH_3_); ^13^C NMR (CD_3_OD, 150 MHz) *δ*_C_ 189.4 (C-9), 183.9 (C-10), 163.6 (C-5), 163.5 (C-1), 138.0 (C-3), 137.3 (C-7), 135.3 (C-9a), 133.8 (C-8a), 131.1 (C-4a), 125.1 (C-6), 120.0 (C-8), 120.0 (C-4), 117.3 (C-10a), 112.7 (C-2), 16.6 (2-CH_3_).

**Compound 10.** C_15_H_10_O_4_. ESI-MS *m*/*z* 253 [M-H]^−^; ^1^H NMR (CD_3_OD, 600 MHz) *δ*_H_ 12.61 (1H, s, 1-OH), 8.31 (2H, d, *J* = 8.0 Hz, H-8,5), 7.85 (2H, t, *J* = 8.0 Hz, H-7,4), 7.69 (1H, t, *J* = 8.0 Hz, H-3), 7.33 (1H, d, *J* = 8.0 Hz, H-2), 4.92 (2H, s, 6-CH_2_OH); ^13^C NMR (CD_3_OD, 150 MHz) *δ*_C_ 188.8 (C-9), 182.4 (C-10), 162.8 (C-1), 147.9 (C-6), 137.0 (C-3), 133.7 (C-4a), 133.5 (C-10a), 133.0 (C-8a), 132.6 (C-7), 128.1 (C-8), 124.7 (C-5), 124.5 (C-2), 119.8 (C-4), 116.4 (C-9a), 64.5 (6-CH_2_OH).

**Compound 11.** C_21_H_18_O_12_. ESI-MS *m*/*z* 461 [M-H]^−^; ^1^H NMR (DMSO-*d_6_*, 500 MHz) *δ*_H_ 7.76 (1H, s, *J* = 2.2 Hz, H-2′), 7.57 (1H, dd, *J* = 8.5, 2.2 Hz, H-6′), 6.80 (1H, d, *J* = 8.5 Hz, H-5′), 6.62 (1H, s, H-3), 6.46 (1H, s, *J* = 2.2 Hz, H-8), 6.12 (1H, s, *J* = 2.2 Hz, H-6), 4.75 (1H, d, *J* = 6.5 Hz, H-1″), 3.16 (1H, s, *J* = 8.5 Hz, H-5″), 2.52–2.48 (10H, m, sugar-H); ^13^C NMR (DMSO-*d_6_*, 125 MHz) *δ*_C_ 181.3 (C-4), 172.7 (C-6″), 165.0 (C-7), 163.7 (C-2), 161.3 (C-5), 157.2 (C-9), 157.2 (C-4′), 146.9 (C-3′), 123.0 (C-6′), 123.0 (C-1′), 117.8 (C-5′), 117.3 (C-2′), 103.7 (C-10), 103.7 (C-3), 103.1 (C-1″), 98.9 (C-6), 94.1 (C-8), 76.0 (C-3″), 73.9 (C-5″), 73.2 (C-2″), 72.0 (C-4″).

**Compound 12.** C_16_H_12_O_5_. ESI-MS *m*/*z* 283 [M-H]^−^; ^1^H NMR (CD_3_OD, 600 MHz) *δ*_H_ 7.98 (1H, s, 8-OH), 7.91 (1H, s, 6-OH), 7.72 (1H, d, *J* = 7.8 Hz, H-4), 7.68 (1H, t, *J* = 8.0, 7.8 Hz, H-5), 7.25 (1H, d, *J* = 8.0 Hz, H-2), 6.80 (1H, overlapped, H-7), 3.88 (3H, s, 1-OCH_3_), 2.36 (3H, s, 3-CH_3_); ^13^C NMR (CD_3_OD, 150 MHz) *δ*_C_ 189.2 (C-9), 183.5 (C-10), 163.2 (C-8), 157.7 (C-6), 153.0 (C-1), 137.4 (C-3), 136.3 (C-4a), 133.7 (C-10a), 127.3 (C-4), 126.5 (C-2), 125.6 (C-9a), 124.4 (C-8a), 119.9 (C-7), 116.9 (C-5), 62.1 (1-OCH_3_), 16.8 (3-CH_3_).

**Compound 13.** C_16_H_10_O_5_. ESI-MS *m*/*z* 281 [M-H]^−^; ^1^H NMR (CDCl_3_, 800 MHz) *δ*_H_ 12.55 (1H, s, 1-OH), 8.46 (1H, d, *J* = 1.6 Hz, H-4), 8.39 (2H, s, H-5,8), 7.87 (1H, dd, *J* = 1.6, 0.9 Hz, H-2), 7.72 (2H, s, H-6,7), 4.02 (3H, s, H-3); ^13^C NMR (CDCl_3_, 200 MHz) *δ*_C_ 187.7 (C-9), 181.8 (C-10), 165.3 (3-COOCH_3_), 162.7 (C-1), 137.0 (C-3), 135.2 (C-6), 135.0 (C-7), 133.3 (C-8a), 133.2 (C-10a), 133.1 (C-4a), 128.3 (C-5), 127.7 (C-8), 124.7 (C-2), 119.8 (C-4), 119.5 (C-9a), 52.8 (3-COOCH_3_).

**Compound 14.** C_15_H_10_O_3_. ESI-MS *m*/*z* 237 [M-H]^−^; ^1^H NMR (CD_3_OD, 600 MHz) *δ*_H_ 8.23 (1H, s, 3-OH), 8.22 (1H, s, *J* = 8.0, 2.0 Hz, H-8), 8.21 (1H, d, *J* = 8.0, 2.0 Hz, H-5), 8.01 (1H, s, H-1), 7.90 (1H, s, *J* = 8.0, 2.0 Hz, H-7), 7.81 (1H, d, *J* = 8.0, 2.0 Hz, H-6), 7.54 (1H, s, H-4), 2.34 (3H, s, 2-CH_3_); ^13^C NMR (CD_3_OD, 150 MHz) *δ*_C_ 184.5 (C-10), 183.7 (C-9), 163.2 (C-3), 135.3 (C-4a), 135.2 (C-7), 134.9 (C-6), 133.8 (C-10a), 131.3 (C-8a), 133.1 (C-2), 130.8 (C-1), 127.9 (C-9a), 127.9 (C-5), 127.0 (C-8), 112.5 (C-4), 16.7 (2-CH_3_).

**Compound 15.** C_29_H_36_O_15_. ESI-MS *m*/*z* 623 [M-H]^−^; ^1^H NMR (CD_3_OD, 500 MHz) *δ*_H_ 7.58 (1H, d, *J* = 15.9 Hz, H-*β*′), 7.05 (1H, d, *J* = 2.0 Hz, H-2′), 6.95 (1H, dd, *J* = 8.2, 2.0 Hz, H-6′), 6.77 (1H, d, *J* = 8.2 Hz, H-5′), 6.66 (1H, d, *J* = 7.8 Hz, H-5), 6.55 (1H, dd, *J* = 7.8 Hz, H-6), 6.28 (1H, s, *J* = 15.9 Hz, H-*α*′), 5.18 (1H, d, H-1″′), 4.37 (1H, d, *J* = 7.5 Hz, H-1″), 2.78 (1H, t, *J* = 7.5 Hz, H-*β*); ^13^C NMR (CD_3_OD, 125 MHz) *δ*_C_ 168.2 (C=O), 149.7 (C-3′), 148.0 (C-*β*′), 146.8 (C-4′), 146.1 (C-4), 144.6 (C-3), 131.4 (C-1), 127.6 (C-1′), 123.2 (C-6′), 121.2 (C-6), 117.0 (C-2), 116.4 (C-5′), 116.2 (C-5), 115.1 (C-2′), 114.6 (C-*α*′), 104.1 (C-1″), 103.0 (C-1″′), 81.6 (C-3″), 76.1 (C-2″), 76.0 (C-5″), 73.7 (C-4″′), 72.3 (C-2″′), 72.2 (C-*α*), 72.0 (C-3″′), 70.5 (C-5″′), 70.4 (C-4″), 62.3 (C-6″), 36.5 (C-*β*), 18.4 (C-6″′).

**Compound 16.** C_9_H_7_NO_2_. ESI-MS *m*/*z* 160 [M-H]^−^; ^1^H NMR (CD_3_OD, 600 MHz) *δ*_H_ 8.08 (1H, s, *J* = 7. 0 Hz, H-8), 7.95 (1H, s, H-2), 7.43 (1H, s, H-5), 7.21–7.15 (2H, m, H-6,7); ^13^C NMR (CD_3_OD, 150 MHz) *δ*_C_ 169.3 (C-8), 138.3 (C-7a), 133.5 (C-2), 127.7 (C-3a), 123.7 (C-6), 122.5 (C-5), 122.1 (C-4), 113.0 (C-7), 108.8 (C-3).

**Compound 17.** C_10_H_10_O_3_. ESI-MS *m*/*z* 177 [M-H]^−^; 1H NMR (500 MHz, CD_3_OD) δ_H_ δ 9.55 (1H, d, *J* = 7.9 Hz, H-9), 7.58 (1H, d, *J* = 15.6 Hz, H-7), 7.23 (1H, d, *J* = 1.9 Hz, H-2), 7.16 (1H, dd, *J* = 8.2, 1.9 Hz, H-6), 6.85 (1H, d, *J* = 8.2 Hz, H-5), 6.63 (1H, dd, *J* = 15.6, 7.9 Hz, H-8), 3.89 (3H, s, 4-OCH_3_); 13C NMR (125 MHz, CD_3_OD) δ_C_ 196.5 (C-9), 156.5 (C-4), 149.4 (C-3), 127.6 (C-8), 126.5 (C-1), 125.2 (C-7), 117.8 (C-6), 116.6 (C-2), 112.1 (C-5), 56.5 (4-OCH_3_).

**Compound 18.** C_42_H_68_O_16_. ESI-MS *m*/*z* 827 [M-H]^−^; ^1^H NMR (DMSO-*d_6_*, 600 MHz) *δ*_H_ 5.24 (1H, s, H-1′), 5.17 (1H, d, *J* = 7.4 Hz, H-12), 4.44 (1H, t, *J* = 7.8 Hz, H-1″), 3.91 (1H, t, *J* = 11.4 Hz, H-2), 3.61 (1H, d, *J* = 3.2 Hz, H-3), 3.49 (1H, s, *J* = 10.4 Hz, Ha-23), 3.43 (1H, t, *J* = 10.4 Hz, Hb-23), 2.25 (1H, m, H-18), 1.22 (3H, d, *J* = 14.3 Hz, H-27), 1.12 (3H, d, *J* = 14.3 Hz, H-25), 0.91 (3H, d, *J* = 5.5 Hz, H-29), 0.85–0.83 (3H, m, H-30), 0.64 (3H, s, H-24), 0.54 (3H, s, H-26); ^13^C NMR (DMSO-*d_6_*, 150 MHz) *δ*_C_ 175.6 (C-28), 138.2 (C-13), 127.0 (C-12), 103.0 (C-1″), 94.0 (C-1′), 76.8 (C-5′), 76.8 (C-2′), 76.7 (C-3′), 76.6 (C-3″), 76.5 (C-5″), 73.5 (C-2″), 72.1 (C-19), 71.6 (C-4″), 69.9 (C-3), 69.9 (C-2), 69.2 (C-4′), 63.8 (C-23), 60.9 (C-6″), 60.9 (C-6′), 53.1 (C-18), 47.4 (C-17), 47.3 (C-9), 46.7 (C-5), 43.1 (C-1), 42.5 (C-4), 42.5 (C-8), 41.2 (C-14), 41.1 (C-20), 37.3 (C-22), 36.6 (C-10), 32.0 (C-7), 28.1 (C-15), 26.4 (C-29), 25.8 (C-21), 24.5 (C-16), 24.2 (C-27), 23.9 (C-11), 16.8 (C-6), 16.7 (C-25), 16.5 (C-26), 16.2 (C-30), 13.7 (C-24).

**Compound 19.** C_20_H_24_O_6_. ESI-MS *m*/*z* 383 [M+Na]^+^; ^1^H NMR (CD_3_OD, 600 MHz) *δ*_H_ 6.95 (1H, s, H-2′), 6.83 (1H, d, *J* = 8.1 Hz, H-6′), 6.76 (1H, d, *J* = 8.1 Hz, H-5′), 6.73 (1H, s, H-4), 6.72 (1H, m, H-6), 5.49 (1H, d, *J* = 6.3 Hz, H-2), 3.86 (3H, s, 7-OCH_3_), 3.82 (3H, s, 3′-OCH_3_), 3.78–3.73 (2H, m, H-3a), 3.57 (1H, s, H-5c), 3.47 (1H, q, *J* = 6.3 Hz, H-3), 2.63 (1H, s, *J* = 7.6 Hz, H-5a), 1.84–1.81 (1H, m, *J* = 7.6 Hz, H-5b); ^13^C NMR (CD_3_OD, 150 MHz) *δ*_C_ 149.2 (C-3′), 147.6 (C-7a), 147.6 (C-4′), 145.3 (C-7), 137.0 (C-5), 134.9 (C-1′), 130.0 (C-4a), 119.8 (C-6′), 118.0 (C-4), 116.2 (C-5′), 114.2 (C-6), 110.6 (C-2′), 89.1 (C-2), 65.1 (C-3a), 62.3 (C-5c), 56.8 (7-OCH_3_), 56.4 (3′-OCH_3_), 55.6 (C-3), 35.9 (C-5b), 33.0 (C-5a).

**Compound 20.** C_22_H_28_O_9_. ESI-MS *m*/*z* 437 [M+H]^+^; ^1^H NMR (CD_3_OD, 500 MHz) *δ*_H_ 7.58 (1H, d, *J* = 15.6 Hz, H-6′), 7.23 (1H, d, *J* = 1.9 Hz, H-2′), 7.16 (1H, dd, *J* = 8.2, 1.9 Hz, H-6), 6.85 (1H, d, *J* = 8.2 Hz, H-7′), 6.63 (1H, dd, *J* = 15.6, 7.9 Hz, H-8′), 4.91 (1H, overlapped, H-7), 4.25 (1H, s, H-9′), 4.19 (1H, s, H-8), 3.91 (6H, s, 3′,5′-OCH_3_), 3.88 (1H, s, H-9b), 3.56 (1H, overlapped, H-9a); ^13^C NMR (CD_3_OD, 125 MHz) *δ*_C_ 155.6 (C-3′,5′), 149.2 (C-3,5), 139.0 (C-4′), 136.2 (C-1), 134.1 (C-4), 133.9 (C-1′), 131.8 (C-7′), 127.4 (C-8′), 105.5 (C-2′,6′), 105.5 (C-2,6), 85.8 (C-8), 73.1 (C-7), 64.1 (C-9′), 62.1 (C-9), 56.9 (3′,5′-OCH_3_), 56.4 (3,5-OCH_3_).

**Compound 21.** C_15_H_20_O_6_. ESI-MS *m*/*z* 295 [M-H]^−^; ^1^H NMR (CD_3_OD, 500 MHz) *δ*_H_ 7.97 (1H, d, *J* = 16.0 Hz, H-4), 6.39 (1H, d, *J* = 16.0 Hz, H-5), 5.84 (1H, s, H-2), 3.84 (1H, m, *J* = 14.3, 6.4 Hz, H-3′ax), 2.26 (1H, dd, *J* = 14.3, 6.4 Hz, H-2′eq), 2.06 (3H, s, 3-CH_3_), 1.89 (1H, dd, *J* = 13.5, 6.4 Hz, H-4′eq), 1.83 (1H, dd, *J* = 14.3, 6.4 Hz, H-2′ax), 1.72 (1H, dd, *J* = 13.5, 11.1 Hz, H-4′ax), 1.34 (3H, s, 1′-CH_3_), 1.07 (3H, s, 5′-CH_3_); ^13^C NMR (CD_3_OD, 125 MHz) *δ*_C_ 181.0 (C-6′), 171.1 (C-1), 148.8 (C-3), 133.4 (C-4), 131.3 (C-5), 122.1 (C-2), 89.8 (C-1′), 82.7 (C-8′), 65.2 (C-3′), 53.4 (C-5′), 42.2 (C-2′), 40.9 (C-4′), 20.9 (3-CH_3_), 18.4 (1′-CH_3_), 14.5 (5′-CH_3_).

**Compound 22.** C_16_H_12_O_5_. ESI-MS *m*/*z* 283 [M-H]^−^; ^1^H NMR (CDCl_3_, 800 MHz) *δ*_H_ 13.77 (1H, s, 5-OH), 13.35 (1H, s, 8-OH), 8.19 (1H, d, *J* = 8.0 Hz, H-4), 8.09 (1H, s, H-1), 7.57 (1H, dd, *J* = 8.0 Hz, H-3), 6.76 (1H, d, *J* = 10.0 Hz, H-6), 2.53 (3H, s, 7-OCH_3_), 1.59 (3H, s, 2-CH_3_); ^13^C NMR (CDCl_3_, 200 MHz) *δ*_C_ 186.6 (C-9), 185.0 (C-10), 156.0 (C-5), 156.0 (C-7), 150.1 (C-8), 145.7 (C-2), 134.6 (C-3), 133.8 (C-4a), 131.5 (C-1a), 131.0 (C-1), 127.0 (C-4), 112.5 (C-9a), 106.5 (C-6), 106.5 (C-10a), 65.5 (2-CH_3_), 22.0 (7-OCH_3_).

**Compound 23.** C_7_H_6_O_5_. ESI-MS *m*/*z* 169 [M-H]^−^; ^1^H NMR (CD_3_OD, 500 MHz) *δ*_H_ 7.05 (2H, s, H-2,6); ^13^C NMR (CD_3_OD, 125 MHz) *δ*_C_ 170.4 (C-7), 146.3 (C-3,5), 139.5 (C-4), 122.0 (C-1), 110.3 (C-2,6).

**Compound 24.** C_30_H_48_O_3_. ESI-MS *m*/*z* 455 [M-H]^−^; ^1^H NMR (CDCl_3_, 500 MHz) *δ*_H_ 5.27 (1H, t, *J* = 3.4 Hz, H-12), 3.21 (1H, dd, *J* = 11.3, 4.3 Hz, H-3), 1.13 (3H, s, H-27), 0.98 (3H, s, H-30), 0.92 (3H, s, H-29), 0.91 (3H, s, H-26), 0.90 (3H, s, H-25), 0.77 (3H, s, H-24), 0.74 (3H, s, H-23); ^13^C NMR (CDCl_3_, 125 MHz) *δ*_C_ 183.4 (C-28), 143.5 (C-13), 122.6 (C-12), 79.0 (C-3), 55.2 (C-5), 47.6 (C-9), 46.5 (C-17), 45.8 (C-19), 41.5 (C-14), 40.9 (C-18), 39.2 (C-8), 38.7 (C-1), 38.3 (C-4), 37.0 (C-10), 33.7 (C-21), 33.0 (C-29), 32.6 (C-7), 32.4 (C-22), 30.6 (C-20), 28.1 (C-23), 27.9 (C-2), 27.4 (C-15), 25.9 (C-27), 23.5 (C-16), 23.4 (C-11), 22.9 (C-30), 18.2 (C-6), 17.1 (C-26), 15.5 (C-25), 15.3 (C-24).

**Compound 25.** C_29_H_50_O. ESI-MS *m*/*z* 437 [M+Na]^+^; ^1^H NMR (CDCl_3_, 500 MHz) *δ*_H_ 5.35 (1H, s, H-6), 3.54–3.51 (1H, m, H-3), 1.00 (3H, s, H-19), 0.92 (1H, d, *J* = 6.6 Hz, H-21), 0.84 (1H, d, *J* = 7.8 Hz, H-29), 0.82 (3H, s, H-27), 0.81 (1H, d, *J* = 6.8 Hz, H-26), 0.68 (3H, s, H-18); ^13^C NMR (CDCl_3_, 125 MHz) *δ*_C_ 140.7 (C-5), 121.7 (C-6), 71.8(C-3), 56.7 (C-14), 56.0 (C-17), 50.1 (C-9), 45.8 (C-24), 42.3 (C-4), 42.3 (C-13), 39.7 (C-12), 37.2 (C-1), 36.5 (C-10), 36.1 (C-20), 33.9 (C-22), 31.9 (C-2), 31.9 (C-8), 31.6 (C-7), 29.1 (C-25), 28.2 (C-16), 26.0 (C-23), 24.3 (C-15), 23.0 (C-28), 21.0 (C-11), 19.8 (C-27), 19.4 (C-21), 19.0 (C-19), 18.7 (C-26), 11.9 (C-18), 11.8 (C-29).

**Compound 26.** C_35_H_60_O_6_. ESI-MS *m*/*z* 577 [M+H]^+^; ^1^H NMR (CDCl_3_, 500 MHz) *δ*_H_ 5.33–5.30 (1H, m, H-6), 4.88 (1H, s, H-1′), 4.20 (1H, m, H-6′), 3.63 (1H, m, H-5′), 3.10 (1H, m, H-4′), 3.04 (1H, m, H-3′), 3.00 (1H, m, H-2′), 0.94 (3H, s, H-19), 0.89 (1H, s, H-29), 0.88 (1H, s, H-27), 0.81(1H, s, H-26), 0.79 (1H, s, H-21_)_, 0.64 (3H, s, H-18); ^13^C NMR (CDCl_3_, 125 MHz) *δ*_C_ 140.4 (C-5), 121.2 (C-6), 100.7 (C-1′), 76.9 (C-3′), 76.7 (C-3), 76.7 (C-5′), 73.4 (C-2′), 70.1 (C-4′), 61.1 (C-6′), 56.1 (C-14), 55.4 (C-17), 49.6 (C-9), 45.1 (C-24), 41.8 (C-13), 38.3 (C-12), 36.8 (C-1), 36.2 (C-10), 35.5 (C-20), 33.3 (C-22), 31.4 (C-7), 31.3 (C-8), 29.2 (C-2), 28.7 (C-25), 27.8 (C-16), 25.4 (C-23), 23.8 (C-15), 22.6 (C-28), 20.6 (C-11), 19.7 (C-26), 19.1 (C-19), 18.9 (C-27), 18.6 (C-21), 11.8 (C-29), 11.7 (C-18).

### 3.2. Antioxidant Activity Assays

**Figure 2:**
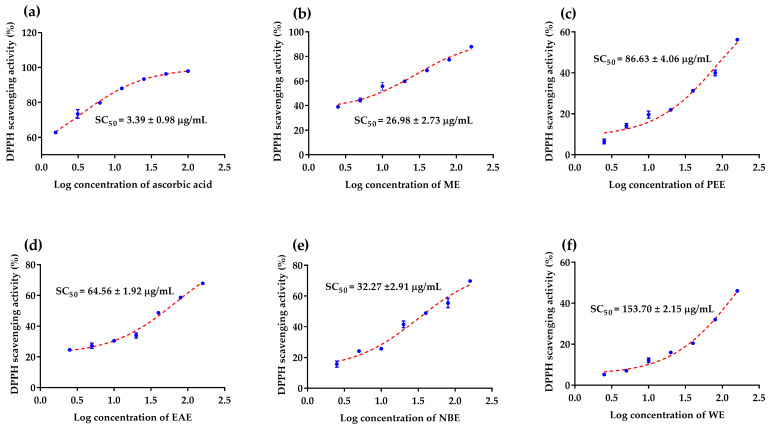
The scavenging effects of *T. grandis* methanolic extract and fractions on DPPH free radicals. (**a**) Log concentration–scavenging rate fitting curve of ascorbic acid. (**b**) Log concentration–scavenging rate fitting curve of ME. (**c**) Log concentration–scavenging rate fitting curve of PEE. (**d**) Log concentration–scavenging rate fitting curve of EAE. (**e**) Log concentration–scavenging rate fitting curve of NBE. (**f**) Log concentration–scavenging rate fitting curve of WF. All values are mean ± SD from three independent experiments. ME: methanolic extract, PEE: petroleum ether extract, EAE: EtOAc extract, NBE: n-BuOH extract and WE: water extract.

Oxidative stress (OS) refers to the imbalance between oxidation and antioxidation in the body. Studies have found that oxidative stress is related to a variety of diseases, such as cardiovascular diseases, diabetes and metabolic disorders [56]. Therefore, the ME, PEE, EAE, NBE, WE and isolated compounds **1**–**23** of *T. grandis* were evaluated for their antioxidant activities according to DPPH, ABTS, and FRAP assays and the half maximal scavenging concentration (SC_50_). As shown in Table 1, the ME had antioxidant activity. This result supported the benefits of using *T. grandis* in herbs and food. The NBE showed significant antioxidant activity, and the EAE showed moderate antioxidant activity. The PEE showed weak antioxidant activity. The WE exhibited no significant antioxidant activity. Furthermore, in the DPPH test, we found that methanolic extract and different fractions were capable of scavenging DPPH free radicals in a concentration-dependent manner (Figure 2). The results showed that the DPPH free radical scavenging ability was ME > NBE > EAE > PEE > WE (Figure 2), and the scavenging capacity of DPPH was related to the accumulative effect of each extract. The results suggested that the main antioxidant compounds of *T. grandis* might present in the NBE and EAE.

The scavenging capacities of compounds **1**–**23** are shown in Table 2. Compounds **13**, **15**, **21** and **23** exhibited significant DPPH antioxidant activities (SC_50_ = 0.32–3.56 µmol/L), compounds **2**, **5**–**8, 15**, **17**, **19** and **23** exhibited significant ABTS antioxidant activities (SC_50_ = 0.8–1.1 mmol Trolox/L) and compounds **5**, **15** and **23** exhibited significant FRAP antioxidant activities (SC_50_ = 1.02–1.22 mmol Trolox/L). These results indicated that compounds **15** and **23** had a potent free radical scavenging ability and ferric reducing power, which might be developed into effective natural antioxidants. In addition, compared with quinones (**9**–**10**, **12**–**14, 22**), flavonoids (**2, 5**–**8**) and phenolic acids (**15**, **17**, **19**, **23**) exhibited stronger ABTS and FRAP scavenging activities. The antioxidant capacities of flavonoids might be related to the degree of methylation. Methylation has been documented to enhance the entry of flavonoids into cells and to prevent degradation, and this might help to enhance antioxidant capacities [57]. Compounds **15** and **23** in *T. grandis* had significant antioxidant capacities, which was consistent with the significant antioxidant capacities of phenolic acids reported in the literature [58]. Interestingly, the potent antioxidant properties of the NBE and EAE might be due to their flavonoid and phenolic ingredients [59], which reminded us that we should pay more attention to the antioxidant activities of polyphenols and flavonoids in *T. grandis*.

### 3.3. Prevention of AAPH-Induced DNA Oxidative Damage Assay

**Figure 3:**
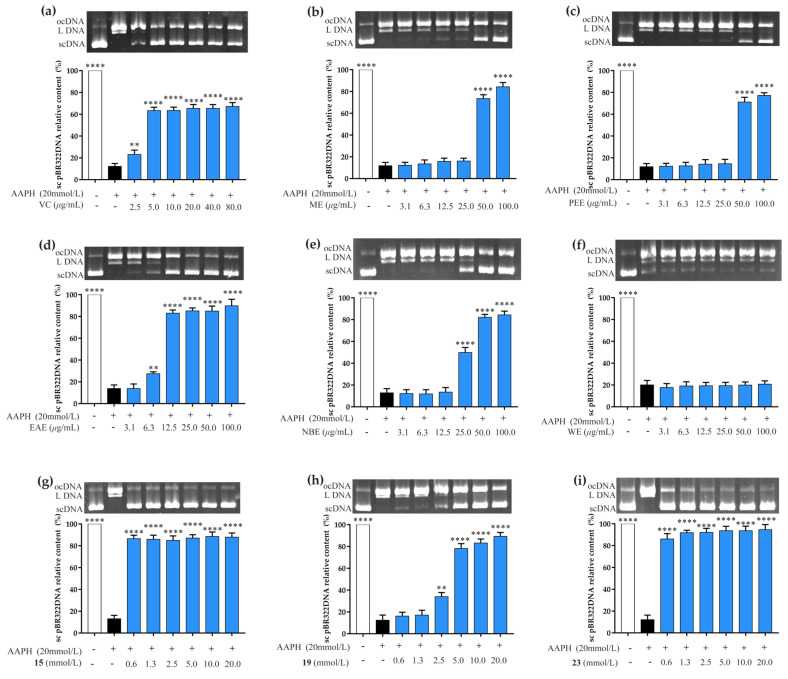
Prevention of 2,2′−Azobis (2−methylpropionamidine) dihydrochloride (AAPH)−induced DNA oxidative damage by *T. grandis* methanolic extract, fractions and compounds **15**, **19** and **23**. (**a**) Prevention AAPH−induced DNA oxidative damage by VC (ascorbic acid). (**b**) Prevention of AAPH−induced DNA oxidative damage by ME. (**c**) Prevention of AAPH−induced DNA oxidative damage by PEE. (**d**) Prevention of AAPH−induced DNA oxidative damage by EAE. (**e**) Prevention of AAPH−induced DNA oxidative damage by NBE. (**f**) Prevention of AAPH−induced DNA oxidative damage by WE. (**g**) Prevention of AAPH−induced DNA oxidative damage by compound **15**. (**h**) Prevention of AAPH−induced DNA oxidative damage by compound **19**. (**i**) Prevention of AAPH−induced DNA oxidative damage by compound **23**. −, inexistent; +, existent. Each group is compared with the model group, significance is denoted by symbols: ** *p* < 0.01, and **** *p* < 0.0001 and all values are mean ± SD from three independent experiments. ME: methanolic extract, PEE: petroleum ether extract, EAE: EtOAc extract, NBE: n-BuOH extract and WE: water extract.

As the most important genetic material in human beings, oxidative damage of DNA can accelerate cell aging and apoptosis, leading to neurodegenerative diseases, inflammation, cancer and other diseases [60,61]. Studies have confirmed that the level of DNA oxidation products in the brain tissues of Alzheimer's patients is significantly increased [62]. DNA is a target for excess oxidative stress, which attacks the bases and sugar moieties, creating strand breaks, altered gene expression and, ultimately, mutagenesis [63]. As shown in Figure 3, when AAPH was added to the model control group, only open circular form (ocDNA) and linear form (linDNA) occurred, resulting in the cleavage of supercoiled circular DNA (scDNA) to ocDNA and linDNA, which indicated that AAPH successfully induced DNA oxidative damage at 20 mM. DNA derived from pBR322 plasmid showed two bands on agarose gel electrophoresis. The faster moving band corresponded to the native form of scDNA and the slower moving band was the ocDNA. The addition of samples to the reaction mixture of AAPH suppressed the formation of linDNA and induced a partial recovery of scDNA.

In this study, Figure 3 shows the electrophoretic pattern of the DNA oxidative damage protection effect induced by AAPH (20 mM) from methanolic extract, different fractions and compounds **15**, **19** and **23** of T. grandis. Compared with the positive control vitamin C, the EAE showed a remarkable protective effect against DNA oxidative damage induced by AAPH. The EAE showed a protective effect at 12.5 μg/mL (*p* < 0.0001) and the NBE showed a protective effect at 25.0 μg/mL (*p* < 0.0001). The ME and PEE began to be protective at 50.0 μg/mL (*p* < 0.0001). The results showed that the protective ability against DNA oxidative damage was EAE > NBE > ME > PEE > WE. Compounds **15**, **19** and **23** showed obvious protective effects against oxidative damage. Compounds **15** and **23** began to have protective effects at 0.6 mM (*p* < 0.0001) and compound **19** began to have a protective effect at 5.0 mM (*p* < 0.0001), which suggests that compounds **15**, **19** and **23** could be developed into effective natural antioxidants. In addition, it is worth noting that the EAE, NBE and compounds **15**, **19** and **23** with significant antioxidant capacities showed significant protective activities against oxidative damage of DNA induced by AAPH, which suggests that the protective effect against DNA oxidative damage might be related to the antioxidant capacities.

### 3.4. Prevention of AAPH-Induced Protein Oxidative Damage Assay

**Figure 4:**
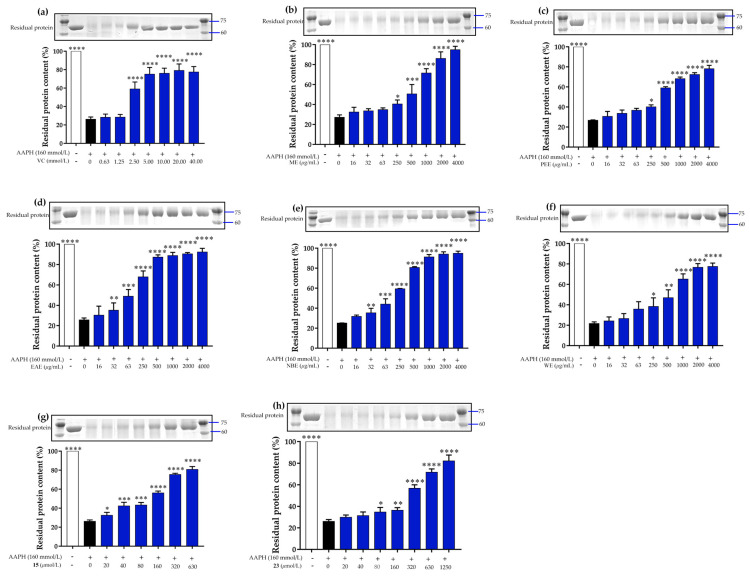
Prevention of AAPH−induced protein oxidative damage by *T. grandis* methanolic extract, fractions and compounds **15** and **23**. (**a**) Prevention of AAPH−induced protein oxidative damage by VC (ascorbic acid). (**b**) Prevention of AAPH−induced protein oxidative damage by ME. (**c**) Prevention of AAPH−induced protein oxidative damage by PEE. (**d**) Prevention of AAPH−induced protein oxidative damage by EAE. (**e**) Prevention of AAPH−induced protein oxidative damage by NBE. (**f**) Prevention of AAPH−induced protein oxidative damage by WE. (**g**) Prevention of AAPH−induced protein oxidative damage by compound **15**. (**h**) Prevention of AAPH−induced protein oxidative damage by compound **23**. −, inexistent; +, existent. Each group is compared with the model group, significance is denoted by symbols: * *p* < 0.05, ** *p* < 0.01, and *** *p* < 0.001, **** *p* < 0.0001 and all values are mean ± SD from three independent experiments. ME: methanolic extract, PEE: petroleum ether extract, EAE: EtOAc extract, NBE: n-BuOH extract and WE: water extract.

The literature reports that AAPH is a free radical generator that can generate alkoxy and alkylperoxy radicals, thereby inducing oxidative damage to macromolecules [64]. Protein degradation and the formation of protein carbonyl groups are the main characteristics of protein oxidation [65,66]. Studies have found that many diseases, such as Alzheimer′s disease, diabetes, cardiovascular and cerebrovascular diseases, are associated with increased protein carbonylation levels [67]. As shown in Figure 4, compared with the control group without AAPH, BSA was induced by AAPH, the BSA bands were noticeably shallower and the carbonylation level of BSA was increased, indicating that hydroxyl free radicals produced by the AAPH induced system can significantly degrade and carbonize BSA. In this experiment, the content of residual protein was used to reflect the abilities of samples to prevent protein oxidative damage.

The results showed that when the concentration of AAPH was 160 mM, the content of residual protein increased with the increase of the sample concentration. As shown in Figure 4d,e, compared with the positive control group (VC), the EAE and NBE showed significant protective effects against protein oxidative damage at 63 μg/mL. In an increased sample concentration, the protective effect became more significant; the EAE and NBE at 1000 μg/mL almost completely inhibited the oxidative damage of BSA induced by AAPH (*p* < 0.0001), the ME and PEE began to show protective effects against protein oxidative damage at 500 μg/mL (*p* < 0.0001) and the WE began to show protective effects against protein oxidative damage at 1000 μg/mL (*p* < 0.0001). The results showed that the protective ability against protein oxidative damage was EAE > NBE > ME > PEE > WE, and this might be related to their better antioxidant capacities. As shown in Figure 4g,h, compounds **15** and **23** have significant protective effects against protein oxidative damage induced by AAPH (160 mM) in a concentration-dependent manner. Compound **15** began to show a protective effect against protein oxidative damage at 40 μM, and compound **23** began to show a protective effect against protein oxidative damage at 320 μM. The results showed that compounds **15** and **23** might be natural antioxidants, which could inhibit the oxidative damage of protein by free radicals.

### 3.5. Anti-Inflammatory Activity Assay

**Figure 5:**
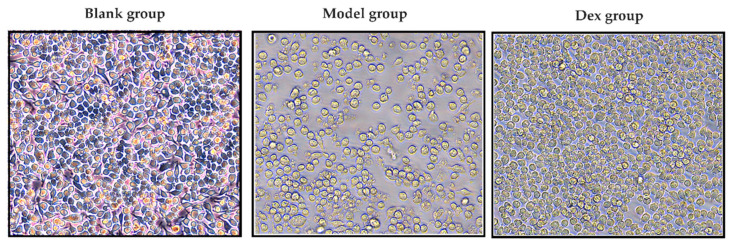
Differentiation and identification of RAW 264.7 cells.

**Figure 6:**
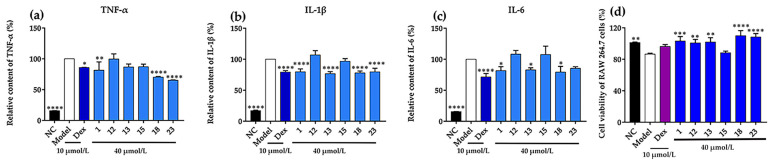
Effect of compounds on cytokine production in RAW 264.7 cells. (**a**) Effect of compounds on TNF-*α* expression in RAW 264.7 cells. (**b**) Effect of compounds on IL-1*β* expression in RAW 264.7 cells. (**c**) Effect of compounds on IL-6 expression in RAW 264.7 cells. (**d**) Cell viability of different groups. The cell viability was compared with the model group for significance testing. All values are mean ± SD from three independent experiments. Significance is denoted by symbols: * *p* < 0.05, ** *p* < 0.01, and *** *p* < 0.001, **** *p* < 0.0001. NC: blank control group, Model: model control group, Dex: positive control group.

LPS is widely used as a stimulator to activate macrophages, inducing the release of pro-inflammatory mediators from macrophages, in particular TNF-*α*, interleukins (IL-1*β*, IL-6), NO and ROS [68]. It is involved in various pathological processes of acute and chronic inflammation. The levels of TNF-*α*, IL-1*β* and IL-6 in macrophage culture supernatants were measured using an ELISA kit, and then the anti-inflammatory effects of compounds **1**, **12**, **13**, **15**, **18** and **23** on LPS-stimulated macrophages were studied. Compared with blank control cells, pro-inflammatory cytokines were increased in the model and positive groups (Dexamethasone) stimulated by LPS (Figure 5). As seen in Figure 6a, compounds **1**, **18** and **23** could decrease TNF-*α* inflammatory factors levels at 40 µM when compared to the model controls (*p* < 0.01). Meanwhile, in Figure 6b, compounds **1**, **13**, **18** and **23** produced a significant decrease in IL-1*β* inflammatory factors levels at 40 µM (*p* < 0.0001). As shown in Figure 6c, compounds **1**, **13** and **18** showed significant activities in reducing the expression of IL-6 inflammatory factors at 40 µM (*p* < 0.05). The anti-inflammatory activity of compound **1** might be related to the fact that the flavonoid was connected by two benzene rings (A and B) through an oxygen-containing heterocycle (C), and with the glycosylation mode on the A ring [69]. Compound **13** has significant anti-inflammatory activity, which was consistent with the literature [4]. The activity of compound **18** might be related to its structure, which is composed of six isoprene and one pentacyclic [70]. The activity of compound **23** might be related to the phenolic hydroxyl groups contained in the structure [71]. The inhibitory effects of compounds **1**, **13**, **18** and **23** on the expression of inflammatory cytokines develop the therapeutic material basis of *T. grandis* in the treatment of inflammation in traditional medicine.

### 3.6. α-Glucosidase Inhibition Assay

**Figure 7:**
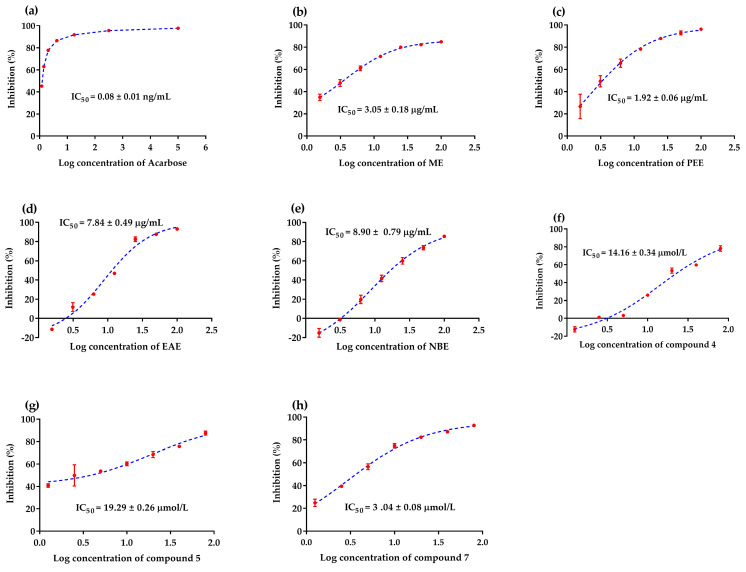
*α*-Glucosidase inhibitory effects of *T. grandis* methanolic extract, fractions and compounds **4**, **5** and **7**. (**a**) Log concentration–inhibition rate fitting curve of acarbose. (**b**) Log concentration–inhibition rate fitting curve of ME. (**c**) Log concentration–inhibition rate fitting curve of PEE. (**d**) Log concentration–inhibition rate fitting curve of EAE. (**e**) Log concentration–inhibition rate fitting curve of NBE. (**f**) Log concentration–inhibition rate fitting curve of compound **4**. (**g**) Log concentration–inhibition rate fitting curve of compound **5**. (**h**) Log concentration–inhibition rate fitting curve of compound **7**. All values are mean ± SD from three independent experiments. ME: methanolic extract, PEE: petroleum ether extract, EAE: EtOAc extract and NBE: n-BuOH extract.

In order to search for active compounds with *α*-glucosidase inhibition, *T. grandis* methanolic extract and different fractions at concentrations of 100, 50, 25, 12.5, 6.25, 3.125 and 1.5625 μg/mL were incubated in 96-well plates for 30 min, and then the *α*-glucosidase inhibition rate was examined. The results are shown in Figure 7b–e. Compared to the positive control acarbose (Figure 7a), the ME and PEE showed the most potent *α*-glucosidase inhibition activities with IC_50_ values of 3.05 ± 0.18 µg/mL and 1.92 ± 0.06 µg/mL, respectively. The EAE and NBE showed moderate *α*-glucosidase inhibition activities with IC_50_ values of 7.84 ± 0.49 µg/mL and 8.90 ± 0.79 µg/mL, respectively. The results indicated that most *α*-glucosidase inhibitory substances might be small polar compounds present in the PEE and EAE. Compounds **1**–**23** were screened for their *α*-glucosidase inhibitory activities, and the results are shown in Table 3. The inhibitory rates of compounds **4**, **5** and **7** on *α*-glucosidase were 68.54%, 69.67% and 85.13%, respectively, whereas compounds **1**–**3**, **6** and **8**–**23** exhibited no significant *α*-glucosidase inhibitory activities, with IC_50_ values greater than 50 µM. Next, the IC_50_ values for compounds **4**, **5** and **7** were measured, and the results are shown in Figure 7f–h. Compound **7** showed the most potent *α*-glucosidase inhibition activity, with an IC_50_ value of 3.04 ± 0.08 µmol/L. Compounds **4** and **5** showed moderate *α*-glucosidase inhibitory activities, with IC_50_ values of 14.16 ± 0.34 µmol/L and 19.29 ± 0.26 µmol/L, respectively. Furthermore, compounds **4**, **5** and **7** had significant *α*-glucosidase inhibitory activities, which might be due to the nucleus of 2-phenylchromone, and the double bond between C2 and C3 in the C ring [72]. Therefore, the *α*-glucosidase inhibitory activities of compounds **4**, **5** and **7** suggested that flavonoids might have better *α*-glucosidase inhibitory activities than the other compounds in *T. grandis*. Our study further provides scientific evidence for the efficacy of *T. grandis* as a herbal medicine in the treatment of diabetes.

### 3.7. α-Glucosidase Enzyme Kinetic Assay

**Figure 8:**
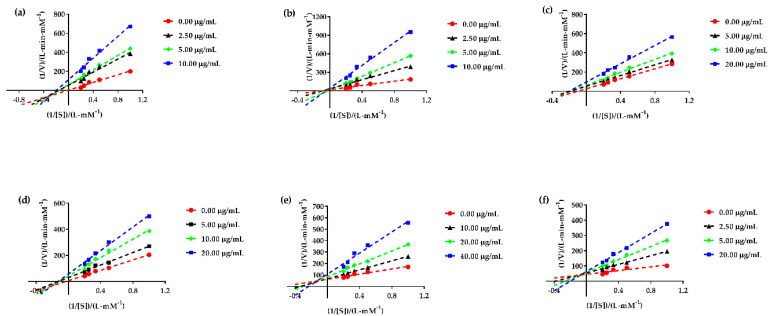
The Lineweaver–Burk plot of *T. grandis* methanolic extract, fractions and compounds **5** and **7.** (**a**) 1/[S]–1/V fitting curve of ME. (**b**) 1/[S]–1/V fitting curve of PEE. (**c**) 1/[S]–1/V fitting curve of EAE. (**d**) 1/[S]–1/V fitting curve of NBE. (**e**) 1/[S]–1/V fitting curve of compound **5**. (**f**) 1/[S]–1/V fitting curve of compound **7**. All values are mean ± SD from three independent experiments. ME: methanolic extract, PEE: petroleum ether extract, EAE: EtOAc extract and NBE: n-BuOH extract.

Lineweaver–Burk plotting was used to evaluate the inhibition type of the methanolic extract, fractions and compounds **5** and **7** partitioned from *T. grandis* on *α*-glucosidase. The concentrations of 1/[*p*NPG] are displayed on the X-axis, and the 1/v values obtained from the Lineweaver–Burk plot are shown along the Y-axis. As shown in Figure 8a–d, all data lines of the ME, PEE, EAE, and NBE on the Lineweaver–Burk plot intersected at a point in the second and third quadrant; with the increasing of inhibitor concentrations, the kinetic parameters of Vmax were decreased, and the kinetic parameters of Km were unchanged. Therefore, all inhibitory effects of samples on the *α*-glucosidase enzyme belonged to the mix inhibition type [73], which suggested that the inhibitors presented in samples ME, PEE, EAE, and NBE might be bound to the enzyme–substrate complex to inhibit *α*-glucosidase. Figure 8e,f show the *α*-glucosidase inhibitory Lineweaver–Burk double count down diagrams for compounds **5** and **7**. *A*ll the data lines of compounds **5** and **7** on the Lineweaver–Burk plot intersected in the second quadrant, with the increasing of inhibitor concentrations, the Vmax of the enzymatic reaction decreased, and the Km values were unchanged, indicating that compounds **5** and **7** were an uncompetitive type of α-glucosidase enzyme inhibition [74]. It is possible to find out the enzyme inhibition effects of the active ingredients through further study of the enzyme reaction, especially of the chemical components which showed significant *α*-glucosidase inhibitory activities in the preliminary screening, so as to provide a valuable active lead for the study of hypoglycemia using *T. grandis*.

### 3.8. Glucose Uptake Activity and Cell Viability Assay

**Figure 9:**
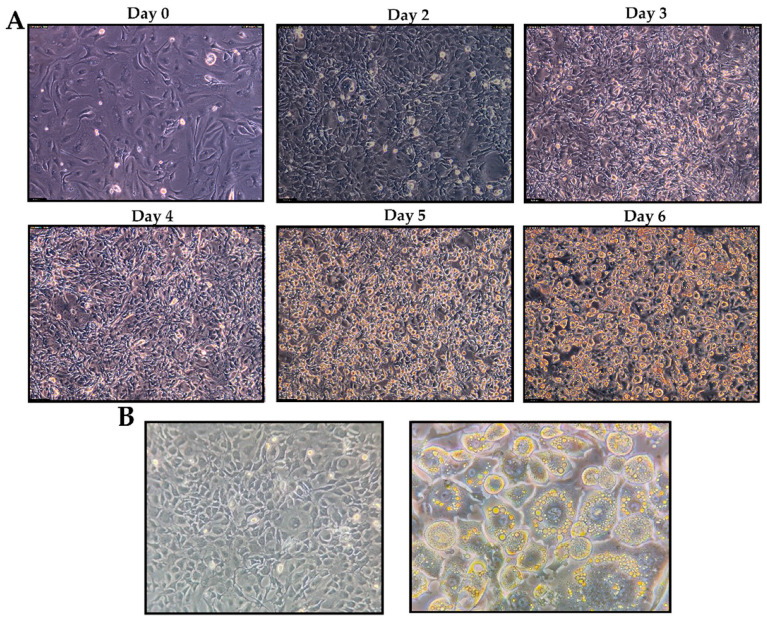
Differentiation and identification of 3T3-L1 adipocytes. (**A**) Differentiation process of 3T3-L1 preadipocytes in the inverted microscope (100×). (**B**) Differentiation process of 3T3-L1 preadipocytes in the inverted microscope (400×).

**Figure 10:**
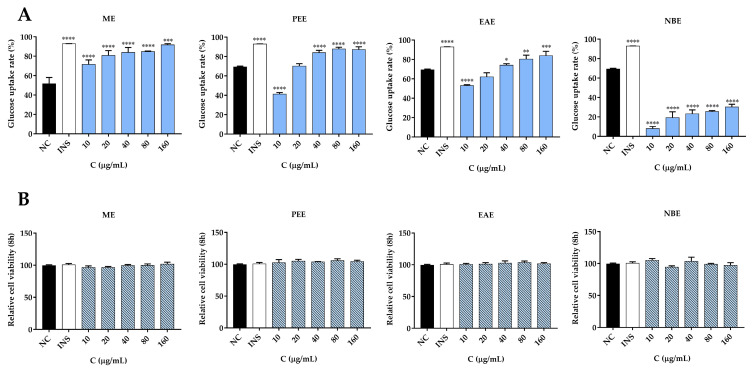
Glucose uptake and cell viability of 3T3-L1 adipocytes treated with *T. grandis* methanolic extract and fractions. (**A**) Glucose uptake rates of different groups (ME, PEE, EAE, and NBE). Insulin and berberine were used as positive controls. (**B**) Cell viability of different groups. All values are mean ± SD from three independent experiments. Significance is denoted by symbols: * *p* < 0.05, ** *p* < 0.01, and *** *p* < 0.001, **** *p* < 0.0001. ME: methanolic extract, PEE: petroleum ether extract, EAE: EtOAc extract and NBE: n-BuOH extract. NC: blank control group, INS: positive control group, 10–160: sampling group.

To examine the glucose uptake activities of methanolic extract, different fractions and the isolated compounds **1**–**23** of *T. grandis*, glucose uptake capacities were evaluated by 3T3-L1 adipocytes. Firstly, the 3T3-L1 preadipocytes were differentiated into mature adipocytes (Figure 9). Next, 10, 20, 40, 80 and 160 μg/mL concentrations of methanolic extract and different fractions were added respectively. After 24 h, the glucose uptake was examined. As shown in Figure 10A, compared with the blank control group, the sample groups of ME, PEE and EAE significantly promoted the glucose uptake rate of 3T3-L1 adipocytes (*p* < 0.0001) and the NBE group significantly inhibited the glucose uptake of 3T3-L1 adipocytes (*p* > 0.0001). The results showed that the main hypoglycemic effect of *T. grandis* might be achieved by improving glucose uptake in 3T3-L1 adipocytes. In addition, the results of the cell viability assay showed that the MEE, PEE, EAE and NBE had no significant effects on the cell viabilities of 3T3-L1 adipocytes (Figure 10B).

**Figure 11:**
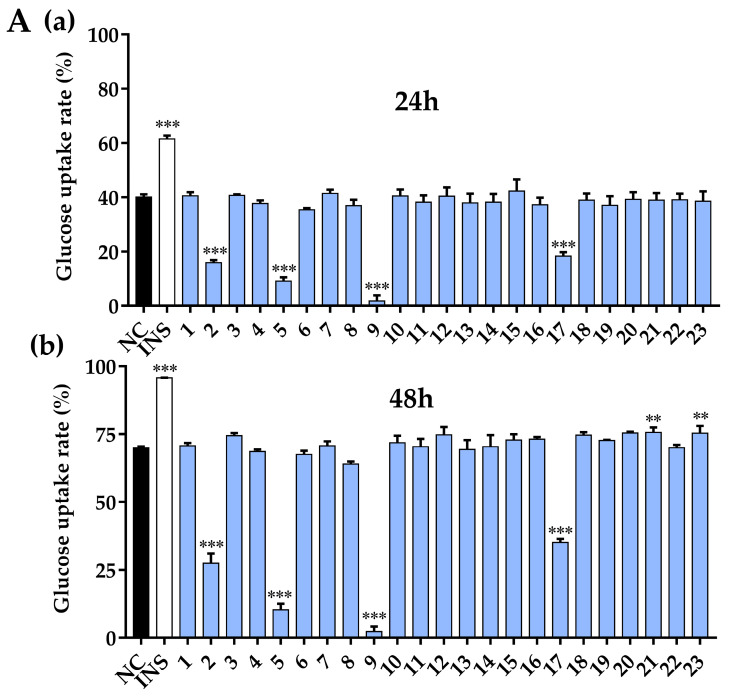

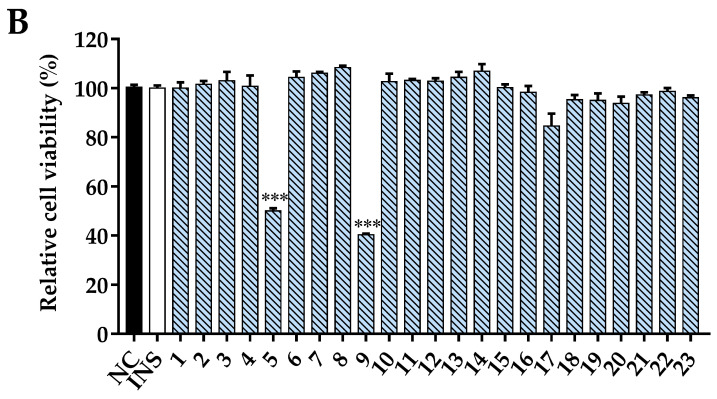
Glucose uptake and cell viability of 3T3-L1 adipocytes treated with *T. grandis* compounds. (**A**) Glucose uptake rate of different groups (**1**–**23**). Insulin and berberine were used as positive controls. (**B**) Cell viability of different groups. All values are mean ± SD from three independent experiments. Significance is denoted by symbols: ** *p* < 0.01, and *** *p* < 0.001. NC: blank control group, INS: positive control group, **1**–**23**: sampling group.

The effects of compounds **1**–**23** on glucose uptake in 3T3-L1 adipocytes were as follows: As shown in Figure 11A(a,b), the results showed that the glucose uptake rate at 40 μM was positively correlated with time, and the glucose uptake rate at 48 h was higher than that at 24 h. As shown in Figure 11A(b), compounds **21** and **23** could significantly promote the glucose uptake of 3T3-L1 adipocytes at 40 µM (*p* < 0.01), and the other tested compounds **1**–**20** and **22** showed no significant promotion effects (*p* > 0.05). Additionally, compared with the blank control, except for compounds **5** and **9,** the positive control group, and all the other tested compounds had no significant effects on the cell viabilities of 3T3-L1 adipocytes (Figure 11B).

## 4. Conclusions

In summary, 26 compounds were isolated from *T. grandis* (EAE, NBE), including nine flavonoids (**1**–**8**, **11**), six anthraquinones (**9**, **10**, **12**–**14**, **22**), one alkaloid (**16**), two pentacyclic triterpenoids (**18**, **24**), four phenylpropanoids (**15**, **17**, **19**, **20**), one sesquiterpene (**21**), phenolic acid (**23**) and two sterols (**25**, **26**). Studies of the antioxidant, anti-inflammatory and anti-diabetic activities of *T. grandis* methanolic extract, fractions and compounds **1**–**23** showed that the EAE, NBE fractions, and compounds verbascoside (**15**), gallic acid (**23**) had remarkable antioxidant activities. Furthermore, compounds verbascoside (**15)** and gallic acid (**23**) showed significantly higher antioxidant activities than the positive control ascorbic acid. Additionally, verbascoside (**15**) and gallic acid **(23)** showed significant protective effects against the oxidative damage of DNA and protein (*p* < 0.001). Simultaneously, the results of inflammatory factor detection showed that luteolin-7-*O*-*β*-D-glucoside (**1)**, 2*β*,3*β*,19*α*,23-tetrahydroxy-urs-12-en-28-*O*-[*β*-D-glucopyranosyl (1-2)-*β*-D-glucopyranosyl] ester (**18**) and gallic acid (**23**) could degrade the expression of inflammatory factors (TNF-*α*, IL-1*β*, IL-6), and 2*β*,3*β*,19*α*,23-tetrahydroxy-urs-12-en-28-*O*-[*β*-D-glucopyranosyl (1-2)-*β*-D-glucopyranosyl] ester (**18**) and gallic acid (**23**) were similar to the positive control Dex inhibitory effects on TNF-*α* and IL-1*β*, which showed significant inhibitory effects on inflammatory cytokines (*p* < 0.001). Meanwhile, ME, PEE fractions, and compounds apigenin-7-*O*-*β*-D-glucuronide methyl ester (**4**), vitegnoside (**5**) and rhamnetin (**7**) had significant *α*-glucosidase inhibition capacities. The enzyme kinetic experiments showed that the inhibitory effects of *T. grandis* methanolic extract, fractions, and compounds vitegnoside (**5**), rhamnetin (**7**) on *α*-glucosidase belonged to mixed and uncompetitive inhibition types. In addition, PEE and EAE significantly promoted the glucose uptake. Our study suggested that the flavonoid and polyphenolic compounds in *T. grandis* might be the major contributors to antioxidant, antiinflammation and *α*-glucosidase inhibition activities. In conclusion, our study enriched the diversity of the chemical composition of *T. grandis*, and provided potential natural candidate drugs to inhibit oxidative stress and related diseases, especially inflammation and diabetes**.**

## Figures and Tables

**Table 1:** Antioxidant activities of methanolic extract and fractions from *T. grandis*.

Samples	DPPH Assay ^A^	ABTS Assay ^B^	FRAP Assay ^C^
	SC_50_ (µg/mL)	mmol Trolox/L	mmol Trolox/L
ME	26.98 ± 2.73 ^d^	0.83 ± 0.02 ^b^	0.58 ± 0.00 ^c^
PEE	86.63 ± 4.06 ^b^	0.91 ± 0.00 ^a^	1.45 ± 0.04 ^b^
EAE	64.56 ± 1.92 ^c^	0.98 ± 0.00 ^a^	2.25 ± 0.04 ^a^
NBE	32.27 ± 2.91 ^d^	0.95 ± 0.01 ^a^	2.70 ± 0.02 ^a^
WE	153.7 ± 2.15 ^a^	0.75 ± 0.01 ^c^	0.48 ± 0.02 ^c^
Ascorbic acid ^A^	3.39 ± 0.98 ^e^	0.99 ± 0.00 ^a^	1.35 ± 0.02 ^b^

Data were expressed as the mean value ± SD (n = 3). Means followed by different superscript letters ^(a–e)^ are significantly different (*p* < 0.05); SC_50_: half scavenging concentration; ^A^ Positive control (DPPH assay, ABTS and FRAP assay); ^B^ The ABTS and ^C^ the FRAP values mean that each gram of sample corresponds to the number of millimoles of Trolox or each mole of sample corresponds to the number of moles of Trolox at the same absorbance. All values are mean ± SD from three independent experiments. ME: methanolic extract, PEE: petroleum ether extract, EAE: EtOAc extract, NBE: n-BuOH extract and WE: water extract.

**Table 2:** Antioxidant activities of compounds **1**–**23** from *T. grandis*.

Compounds	DPPH Assay ^A^	ABTS Assay ^B^	FRAP Assay ^C^
	SC_50_ (µmol/L)	mmol Trolox/L	mmol Trolox/L
**1**	8.09 ± 1.05 ^c^	0.08 ± 0.01 ^d^	n.d.
**2**	17.38 ± 1.08 ^b^	0.92 ± 0.01 ^a^	0.03 ± 0.01 ^c^
**3**	5.22 ± 0.19 ^cd^	0.05 ± 0.00 ^d^	0.01 ± 0.01 ^c^
**4**	31.52 ± 1.75 ^a^	0.21 ± 0.01 ^b^	0.02 ± 0.00 ^c^
**5**	9.92 ± 0.36 ^c^	0.92 ± 0.00 ^a^	1.02 ± 0.01 ^a^
**6**	22.12 ± 1.24 ^a^	0.99 ± 0.07 ^a^	0.37 ± 0.05 ^b^
**7**	16.94 ± 0.67 ^b^	0.92 ± 0.07 ^a^	0.33 ± 0.04 ^b^
**8**	n.d	1.05 ± 0.01 ^a^	0.01 ± 0.00 ^c^
**9**	n.d	n.d	n.d
**10**	n.d	0.13± 0.00 ^c^	0.01 ± 0.01 ^c^
**11**	n.d	0.65 ± 0.00 ^b^	0.01 ± 0.00 ^c^
**12**	n.d	0.03 ± 0.00 ^d^	n.d
**13**	3.56 ± 0.53 ^d^	0.04 ± 0.00 ^d^	0.01± 0.01 ^c^
**14**	25.77 ± 0.57 ^a^	0.09 ± 0.06 ^d^	0.03 ± 0.00 ^c^
**15**	1.99 ± 0.34 ^d^	1.10 ± 0.77 ^a^	1.22 ± 0.11 ^a^
**16**	12.31 ± 0.53 ^b^	0.78 ± 0.01 ^a^	0.28 ± 0.01 ^b^
**17**	n.d	0.80 ± 0.01 ^a^	n.d
**18**	8.45 ± 0.57 ^c^	n.d	0.39 ± 0.00 ^b^
**19**	6.17 ± 0.06 ^c^	0.85 ± 0.03 ^a^	0.25 ± 0.01 ^b^
**20**	8.66 ± 0.43 ^c^	0.67 ± 0.01 ^b^	0.29 ± 0.00 ^b^
**21**	2.61 ± 0.26 ^d^	0.13 ± 0.01 ^c^	0.02 ± 0.00 ^c^
**22**	n.d	0.32 ± 0.01 ^bc^	n.d
**23**	0.32 ± 0.07 ^e^	0.95 ± 0.02 ^a^	1.03 ± 0.01 ^a^
Ascorbic acid ^A^	13.45 ± 0.02 ^b^	0.85 ± 0.00 ^a^	1.01 ± 0.06 ^a^

Data were expressed as the mean value ± SD (n = 3). Means followed by different superscript letters ^(a–e)^ are significantly different (*p* < 0.05); SC_50_: half scavenging concentration; ^A^ Positive control (DPPH assay, ABTS and FRAP assay); ^B^ The ABTS and ^C^ the FRAP values mean that each gram of sample corresponds to the number of millimoles of Trolox or each mole of sample corresponds to the number of moles of Trolox at the same absorbance. Positive control (ascorbic acid); n.d., not determined. All values are mean ± SD from three independent experiments.

**Table 3:** *α*-Glucosidase inhibitory activities of compounds **1**–**23** from *T. grandis*.

Compounds	Inhibitory Rate (%) ^A^	IC_50_ (µmol/L)
**1**	10.62 ± 1.39 ^d^	>50.00
**2**	31.35 ± 6.46 ^c^	>50.00
**3**	29.06 ± 1.86 ^cd^	>50.00
**4**	68.54 ± 2.90 ^b^	14.15 ^a^
**5**	69.67 ± 2.20 ^b^	19.29 ^a^
**6**	12.96 ± 2.08 ^d^	>50.00
**7**	85.13 ± 0.81 ^ab^	3.04 ^b^
**8**	1.27 ± 0.25 ^e^	>50.00
**9**	3.13 ± 4.06 ^e^	>50.00
**10**	1.77 ± 4.08 ^e^	>50.00
**11**	4.57 ± 4.37 ^e^	>50.00
**12**	53.04 ± 0.36 ^bc^	>50.00
**13**	13.84 ± 0.93 ^d^	>50.00
**14**	20.03 ± 2.44 ^c^	>50.00
**15**	18.30 ± 2.22 ^cd^	>50.00
**16**	12.53 ± 0.76 ^d^	>50.00
**17**	8.69 ± 1.94 ^de^	>50.00
**18**	22.91 ± 1.10 ^c^	>50.00
**19**	57.40 ± 1.32 ^bc^	>50.00
**20**	40.30 ± 0.76 ^c^	>50.00
**21**	21.41 ± 4.01 ^c^	>50.00
**22**	20.58 ± 1.30 ^c^	>50.00
**23**	21.89 ± 3.03 ^c^	>50.00
Acarbose	99.53 ± 0.01 ^a^	0.062 ^c^

Data were expressed as the mean value ± SD (n = 3). Means followed by different superscript letters ^(a–e)^ are significantly different (*p* < 0.05); IC_50_: half inhibition concentration; ^A^ Percent inhibition at a concentration of 50 µM; IC_50_ > 50.00, not determined.

## Data Availability

Not applicable.
